# No Self Without Salience: Affective and Self-relevance Ratings of 552 Emotionally Valenced and Neutral Dutch Words

**DOI:** 10.1007/s10936-021-09784-1

**Published:** 2021-06-14

**Authors:** Lora I. Dimitrova, Eline M. Vissia, Hanneke Geugies, Hedwig Hofstetter, Sima Chalavi, Antje A. T. S. Reinders

**Affiliations:** 1 Department of Psychosis Studies, King’s College London, London, UK; 2 Heelzorg, Centre for Psychotrauma, Zwolle, The Netherlands; 3 University Centre of Psychiatry, University Medical Center Groningen, University of Groningen, Groningen, The Netherlands; 4 Department for Research, Information and Statistics, Municipality of Amsterdam, The Netherlands; 5 Movement Control and Neuroplasticity Research Group, Department of Movement Sciences, KU Leuven, Leuven, Belgium; 6 Department of Psychological Medicine, Institute of Psychiatry, Psychology & Neuroscience, King’s College London, London, UK; 7 Department of Psychiatry, Amsterdam UMC, Location VUmc, VU University Amsterdam, Amsterdam, The Netherlands

**Keywords:** Concept of self, Self-relevance, Valence, Standardized stimulus set, Dutch

## Abstract

It is unknown how self-relevance is dependent on emotional salience. Emotional salience encompasses an individual's degree of attraction or aversion to emotionally-valenced information. The current study investigated the interconnection between self and salience through the evaluation of emotional valence and self-relevance. 56 native Dutch participants completed a questionnaire assessing valence, intensity, and self-relevance of 552 Dutch nouns and verbs. One-way repeated-measures ANCOVA investigated the relationship between valence and self, age and gender. Repeated-measures ANCOVA also tested the relationship between valence and self with intensity ratings and effects of gender and age. Results showed a significant main effect of valence for self-relevant words. Intensity analyses showed a main effect of valence but not of self-relevance. There were no significant effects of gender and age. The most important finding presents that self-relevance is dependent on valence. These findings concerning the relationship between self and salience opens avenues to study an individual's self-definition.

**Supplementary Information:**

The online version contains supplementary material available at 10.1007/s10936-021-09784-1.

## Introduction

Interest among researchers in the concept of self and self-relevance is growing (e.g. Crocetti et al., 2015). However, it has been difficult to find a clear and accurate definition for ‘the concept of self’. Several different terminologies, such as ‘self-awareness’, ‘self-relevance’, ‘self-consciousness’ and ‘self-image’, have been proposed to clarify the understanding of the self. Definitions come with issues such as the idiosyncrasy of the nature of the meaning, which makes measurement very difficult to implement. Weiss and Cropanzano (1996) suggest that at the core of all cognitive appraisal theories lies an initial evaluation based on relevance to well-being, that is self-relevance, presented in simple positive or negative terms. This evaluation influences the intensity of the emotional reaction towards the stimuli, which in turn leads to further, more specific appraisals. In the field of emotion research, certain aspects of self-relevance can be investigated by the use of standardized, validated affective stimuli sets, e.g. pictures (Gruhn & Scheibe, 2008), sounds (Bradley & Lang, 2000) or words (Bradley & Lang, 1999; Fields & Kuperberg, 2016). However, these stimuli are usually obtained in an English setting and their applicability to other languages and cultures remains unknown.

Although language provides researchers with the richest possibility for nuances, word validation studies present a language dependent challenge. The relationship of verbs and their valence and self-relevance is lacking in empirical evidence, hence the need to examine such stimuli with regard to emotional connectivity and relevance is eminent. The advantage of using word stimuli as compared to other types of emotional stimuli, such as pictures, is the possibility of using certain word nuances whose meaning is not easily captured in one single picture. However, small nuances of words, due to the translations, can cause a large difference in the meaning of a specific word. Word sets for a different language cannot be created simply by translating these validated words as this would violate the original high validity scores. For Dutch words, standardized affective stimulus sets studies such as Hermans and De Houwer (1994) and Van Rensbergen et al. (2015), provide ratings on separate factors like affect (positive and negative) and familiarity. Unfortunately, Hermans and De Houwer (1994) did not report any interaction effects between affect and familiarity, while Van Rensbergen et al. (2015) did not assess for self-relevance, hence it is not concluded how emotional connection is linked to self-relevance of words. In addition, the generalizability of the study by Hermans and De Houwer (1994) is unknown due to small language differences between the Netherlands and Belgium, therefore, the factor ‘familiarity’ might be a problematic concept when making a transfer to the Dutch language. Furthermore, Van Rensbergen et al. (2015) made use of computationally estimated word covariates, which can vary when compared to human judgement. When presented with the issue of lack of validated word databases, studies have been using words without properly validating them (e.g. Thomaes et al., 2009), which further highlights the need to contribute to existing evidence and generate validated word sets to be used in future research.

In terms of valence, negative and neutral valenced stimuli, have often been deemed as less important than positive counterparts in such research, producing less contrasting effects (e.g. Agustí et al., 2017). Nevertheless, the importance of negative stimuli should not be overlooked, as negative affect evokes stronger more rapid, physiological, cognitive, emotional, and social response in individuals, compared to neutral and positive (Cacioppo et al., 1999). Negative affect is showcased through the way an individual relates to the negative information presented (Sauer-Zavala et al., 2012), which might be mediated by the degree of self-relevance. Valence is responsible for attributing stronger emotional connectivity to statements (Bromgard et al., 2006), and therefore, stronger self-relevance ratings. However, of issue lies the observed positivity bias in healthy populations, as individuals remember or look for information that positively affects them, or they find is associated with them (Mezulis et al., 2004), overshadowing the importance of negative and neutral stimuli.

The semantic differential theory, devised by Osgood et al. (1957), indicating that meaning of words is appraised on three reoccurring dominant dimensions of adjectival characteristics (evaluation, activity and potency), which correspond to fundamental psychological attributes and to the organisation of processes. ‘Evaluation’ refers to the tendency to approach, or avoid, a stimulus with regard to its negative or positive reinforcement, and is measured depending on the conceptual domain (Carroll et al., 1959). ‘Activity’ is the necessity, or non-necessity, of performing an action with regard to making an adjustment to the stimuli. ‘Potency’ is the measure of the amount of effort exerted into the response to the adjustment (Carroll et al., 1959). Badia et al. (2014) suggest that the semantic differential technique may be the best choice for measuring an individual’s identity meaning related to emotions. Through the sematic differential meaningful response to the self as an object can be captured namely, using the semantic differential components in research allow to objectively measure and understand the self better (Burke & Stets, 2009). Ho et al. (2015) indicate that three components of emotion, comparable to the components of the semantic differential theory, have typically been studied in word appraisal research: valence (positive or negative feelings triggered by the stimuli; the degree of pleasantness, or unpleasantness of stimuli) arousal (the alertness, attentiveness or excitement a stimulus evokes, i.e. intensity of a stimulus), and dominance (or degree of control exerted by the stimuli i.e. the stimulus’ relevance to the self). Consequently, measuring an individual’s response with regard to valence, intensity and self-relevance can provide valuable insight into their self.

Interestingly, emotional appraisals have been found to be influenced by the gender of the appraiser. Hoffmann et al. (2010) found that women show a small to moderate advantage in emotion recognition, particularly at lower intensities, but no differences between the genders on higher intensity levels of emotional charge. Such findings were also confirmed in a recent meta-analysis by Fischer et al. (2018), who found evident female advantage in detecting subtle emotional ques. Women, compared to men, also use more extreme ratings of pleasantness (Bellezza et al., 1986). On the other hand, there is recent evidence which poses that gender exerts no influence over valence allocation (Ho et al., 2015), which emphasises the need to examine the relationship further.

Furthermore, age has also been found to influence word ratings. Grühn and Smith (2008) showed that younger and older adults agreed on whether a word is more or less positive than another word, but age groups differed in their exact evaluations of how positive or negative these words were rated. The positivity bias refers to searching for information that is positively related to the individual, and confirms positive qualities the individual beliefs to possess. Particularly, children and older adults display the largest positive bias towards information (Mezulis et al., 2004). Previous studies (Bellezza et al., 1986; Bradley & Lang, 1999; Janschewitz, 2008) included university students as samples. However, such samples usually consist of young, diverse, liberal and open-minded students (Janschewitz, 2008), and are likely to be unrepresentative of the general population.

It is important to expand the realm of knowledge on emotional connectivity to words, including in the Dutch language. The present word study assesses valence, self-relevance and intensity, and has several aims. The first aim was to investigate whether valence is dependent on the self-relevance rating of a word. More specifically, it was aimed to investigate whether words rated by participants as negative were rated as more self-relevant than neutral words. The second aim was to study the effects of age and gender on word evaluations. We hypothesized (i) that the self-relevance of a word is dependent on the valence of a word, (ii) that a word rated as ‘self-relevant’ will also receive a higher intensity score, and (iii) that valence ratings are gender and age dependent.

## Methods

### Participants

The participants were acquired from the general population through opportunity sampling by the means of voluntary recruitment and local advertisement. Only people above the age of 18 were included. Data from 56 native Dutch participants was entered in the analyses, 31 women (55.35%) and 25 men (44.64%), of varying ages from 24 to 62 years old (*M* = 40.03, *SD* = 12.61). Education level was also obtained, presenting varying results from low, medium and high class, with high education class being most prevalent (67.85%) (Table 1). Oral informed consent was obtained at the start of the study. Ethical approval had been obtained and performed in accordance to Dutch law. The current study was part of a larger neuroimaging study, for which overall approval from the Medical Ethical Committee of the University Medical Center Groningen for research was obtained (Reference number: METC2008.211). Throughout the process, data remained confidential and anonymous, and the participants were aware of their right to withdraw at any time during the process.

**Table 1:** Demographic characteristics of the population

Characteristics	N (%)
Total	56 (100%)
Male	25 (44.64%)
Female	31 (55.35%)
Age distribution	
20–30 years	22 (39.28%)
30–40 years	13 (23.21%)
40–50 years	12 (21.42%)
> 51 years	9 (16.07%)
Education class	
Low education class	5 (8.92%)
Medium education class	13 (23.21%)
High education class	38 (67.85%)

### Stimulus Selection

The current study only sampled neutral and negative words, positive valenced words were not sampled for this study. Our main interest was to investigate self-relevance of neutral and negative words, with which to create representative words sets to be used in future research. We also had to limit the length of the wordlist to increase the likelihood that subjects completed the rating of all words.

#### Neutral Words

Neutral nouns were adopted from a study by Hermans and De Houwer (1994). The words were rated on a 7-point Likert scale, beginning from ‘very negative’ (‘1’) and leading to ‘very positive’ (‘7’). From this study, nouns with an affective mean rating between 3.50 and 4.50 (neutral) were taken into account. Additionally, nouns and verbs were adopted from a list that was used for the Deese/Roediger-McDermott Paradigm in a study by Geraerts et al. (2005). Furthermore, neutral verbs and nouns were adopted from a study by Ter Laak (1992). From this latter study, only words with a result of a z-score of 0.82 or higher on neutral allocation, and not indicated as ‘happy’, ‘sex’, ‘sad’, or ‘fearful’ were selected, as those propose a positive or negative impression.

#### Negative Words

To select the negative words, nouns with a very or modestly negative (‘1’ and ‘2’ on the Likert scale) affective rating in a study by Hermans and De Houwer (1994) were included. Furthermore, verbs and nouns as indicated of being negative or traumatic used in a study by Thomaes et al. (2009) were integrated. Verbs and nouns rated as unpleasant in a study by Tops et al. (2003), were also selected. Additionally, verbs and nouns were selected if they were rated as having a high score on ‘fear’, ‘sadness’, or ‘sex’ in the study by Ter Laak (1992). Words from this latter study were selected if they had a z-score of 0.90 or higher on either ‘sex’, ‘sad’ or ‘fearful’, and no score on the other emotional ratings. This meant that only words with negative connotation were approved. Moreover, words were adopted from a list that was used for the Deese/Roediger-McDermott Paradigm in a study by Geraerts et al. (2005).

#### Criteria

Overall 4241 words were identified and selected from the above-mentioned studies. Selection criteria were applied to reduce the number of words to rate to a more manageable amount. With the idea to include words of similar lengths and syllables, first the word length had to be between five and nine characters. This range was composed using the mean number of letters plus or minus one standard deviation. Second, words had to consist of only one, two, or three syllables. Third, words which appeared as both nouns and verbs in the neutral or negative wordlists were removed from the final list. This was done in order to reduce response error due to confusion of accuracy of choice. Finally, 552 words were deemed acceptable (see Appendix A), half of which offered a negative connotation, the other half, neutral. Three versions of the word-sets were then created with different order of all of the words to be presented to the participants. They consisted of both negative and neutral stimuli, randomly assigned, in order to correct for boredom and repetition effects.

### Procedure

Participants rated the 552 emotionally valenced words, 425 nouns (77%) and 127 verbs (23%) presented on a computer in the form of a questionnaire, spread across multiple pages. Participants were first asked to rate the valence felt towards a word in a multiple-choice format consisting of five options:

1. Positive;
2. Neutral;
3. Negative;
4. Both positive and negative;
5. I cannot decide.

By including ‘positive’, ‘both positive and negative’, and ‘I cannot decide’ selection options, we prevented limited rating choices and uncontrollable factors, such as demand characteristics. As such, this enables capturing a genuine emotional connotation. It was important to provide adequate selection options to avoid incorrect categorisation of words, as well as contamination of validated word sets. The rating of truly negative and truly neutral could be ensured, assuming positive ratings were allocated to seemingly ambiguous stimuli not adherent to the negative or neutral category, or to false-neutral words derived from the initial studies.

Consequently, if the selections involved positive or negative connotations, the intensity of the feeling was assessed on a 4-point scale ranging from:

1. Not intense;
2. Slightly intense;
3. Intense;
4. Very intense.

Valence and intensity were assessed dependently, as a set, meaning intensity was only allocated if the word was scored to have either a positive or a negative valence. Neutral intensity was not assessed because intensity is more strongly associated with an emotionally charged stimuli (Brosch et al., 2009). Neutral valence does not evoke the strong emotional reaction negative and positive valence do, so if intensity was assessed, it would undermine the reliability of the allocations of the emotional words. This can overestimate the effects of emotion felt on neutrally valenced words, and underestimate effects on the emotionally charged words (Hofmann et al., 2009). In this line of argument, the exclusion of intensity allocations on neutral words was further justified.

Second, self-relevance towards the word was assessed on a 3-point Likert scale indicating options:

1. Not related to me;
2. A little related to me;
3. Very related to me.

Self-relevance was measured on three options to reduce the level of confusing information and complications of choice for the participants, which could potentially alter the accuracy of their rating. The idiosyncrasy of experience which shapes such an assessment would implicate the creation of sets of negative and neutral words, and explains why the questionnaire options were required to be as concise and straightforward as possible, resulting in less individual differences affecting the results. Both self-relevant and non-self-relevant words were included in the analyses, first as a grouped variable and then as two different variables depending on the allocation of either self-relevance or no self-relevance to examine any effects.

### Statistical Analysis

#### Variables

The first hypothesis, indicating that self-relevance is dependent on the valence of a word, has the self-relevance rating as the dependent variable (DV) and the different allocated valence as the independent variable (IV). The second hypothesis, regarding that a word rated as ‘self-relevant’ will receive a higher intensity rating, has as IV the self-relevance of a word, while the DV is the intensity allocation. The IVs for the final hypothesis, which is that valence ratings are gender and age dependent, are the ages and the gender of the participants, while the DV is the valence of a word.

#### Reliability

To be certain that the outcomes represent valuable information, reliability was calculated using Cronbach’s alpha on the valence and self-relevance scales. This was performed on a separate dataset, involving all ratings to every word. The intensity scale was omitted due to the high number of missing values caused by the absence of intensity assessment of neutral words. Validation tests were also performed on the words and participants. The maximum missing cases was set at 70%, and the coefficient of variation at < 0.001. In doing so words which failed these cut-offs were removed from the final sets of validated negative and neutral words, assuring higher level of reliability.

#### Analysis

The interaction between valence and the self was assessed through analyses of ratings on the three valences (positive, negative and neutral) and self-relevance (self-relevant, non-self-relevant) allocations. To deal with the issue of the ‘a little related to me’ option, the individual self-relevance ratings of the words were averaged. A value between 1 and 1.49 was representative of a non-self-relevant allocation, while a value between 1.50 and 3—self-relevant. The data were then distributed with the total count of items per category based on valence and self-relevance into neutral self-relevant, neutral non-self-relevant, negative self-relevant, negative non-self-relevant, positive self-relevant and positive non-self-relevant variables for each participant. The data was examined for skewness, and following deviations from normality, we have performed logarithmic (log10) transformations to ensure normal distributions across the scales. To assess the dependency of valence on self-relevance, one-way repeated-measures analysis of covariance (ANCOVA) was used on the self-relevant portion of the words (neutral, negative and positive self-relevant variables). To determine the effects of gender and age, gender was placed as a between-subject factor, while age as a covariate. Additional pairwise comparisons were also performed. Cohen’s d was calculated for the post-hoc pairwise comparisons by dividing the adjusted mean difference by the square root of the Mean square from analysis of variance (Howell, 2010), and was interpreted using Cohen’s (1988) benchmark of small (0.2), medium (0.5) and large (0.8).

Intensity analyses were then performed for negative and positive valenced words only. The mean intensity rating for each participant was computed using the count of items from the four rating options, and it was divided based on valence and self-relevance into: mean positive self-relevant intensity rating; mean positive non-self-relevant intensity rating; mean negative self-relevant intensity rating; mean negative non-self-relevant intensity rating. Log10 transformations were performed to ensure a normal distribution of the data. A 2 (positive, negative) × 2 (self-relevant, non-self-relevant) repeated-measures ANCOVA was performed to determine any effects of valence and self-relevance on intensity ratings, including gender as a between-subjects factor and age as a covariate. Pairwise comparisons were performed, and Cohen’s d was calculated as above.

#### Word Analyses

It was additionally aimed to devise sets of negative and neutral words from the previously obtained data, which could be used in future appraisal studies. To do this the mode was calculated for each word with the numbers representing a category of valence allocated (1 = positive; 2 = neutral; 3 = negative; 4 = both positive and negative; 5 = I cannot decide), as per valence rating options (see Procedure). The valid percentage of the number of subsequent valence allocation per category was used, to eliminate the influence of missing values. The highest valid percentage was taken into account as representative. In order for a word to represent a valence, words allocated as concrete options of ‘positive’ ‘negative’ or ‘neutral’ (options a, b, or c in methods) needed to have a valid percentage of above 70% to comprise of a definite majority with at least 39 individuals from 56 participants agreeing. Words which fell under the ‘both positive and negative’, ‘I cannot decide’ or had multiple concrete allocations, the valid percent needed to be above 50%, meaning at least 28 participants rated the word the same way, which constituted half of the total of the participants. This was chosen due to the varying levels of agreement on the word allocation on behalf of the participants, while still maintaining the integrity for the majority of ratings being representative of a valence. Any multi-allocated words below the selection criteria of above 50%, or single-allocated words with a valid percent of below 70%, were not included in the finalised sets. Additionally, any words which were indicated as failing the validity checks of having more than 70% missing cases and/or a significant variation outcome, were removed from the lists. The percentage of missing cases was only considered for the positive and negative valenced words.

## Results

### Reliability and Validity

Out of the 552 words, 20 were deemed unsuitable for reliability testing due to missing values and were omitted from the analysis, which resulted in a final of 532 items on the valence scale being examined, obtaining a very high reliability rating of *α* = 0.967. The self-relevance scale obtained an alpha of *α* = 0.987, with 548 items examined and an exclusion of four words for missing data. Appendix B presents the missing words from the reliability tests. The valence scale indicated that six words failed the validation tests for the validity assessment, obtaining a coefficient of variation of < 0.001. The intensity scale indicated 96 words as having more than 70% missing cases, which were the neutral words, while eight words failed the validation test. The self-relevance scale displayed that two words failed the validation test and obtained a coefficient of variation of < 0.001 (see Appendix C).

### Main effects and Interaction Effects

#### Self-relevance and Valence

**Fig. 1:**
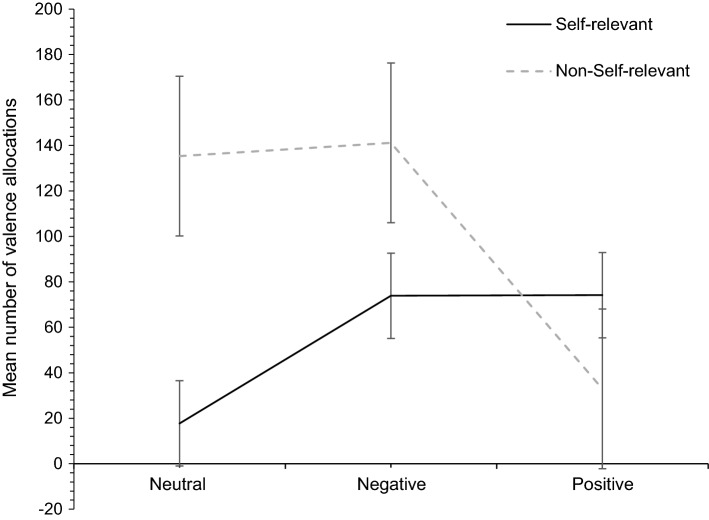
A depiction of the mean number of self-relevance and valence allocations

One-way repeated-measures ANCOVA presented a main effect of valence (*F*(1.637, 80.194) = 16.011, *p* < 0.001, n_p_^2^ = 0.246), and no interaction effects of gender and age. Pairwise comparisons indicated a difference between neutral and negative valence (*p* < 0.001), with a mean difference of 0.762 in favour of greater self-relevance being allocated to negative words and a medium effect size of *d* = 0.54. There was also a difference between neutral and positive valence (*p* < 0.001), with a mean difference of 0.810 in favour of allocating greater self-relevance to positively rated words and a medium effect size of *d* = 0.57. There was no significant difference between self-relevant positive and negative valence, indicating self-relevance to be equally as allocated for both valences. Figure 1 depicts the relationship between self-relevance and valence using the means of self-relevant versus non-self-relevant positive, neutral and negative valence. There are more emotional self-relevant words, as compared to neutral words. Neutral and negative words were also more often rated as non-self-relevant as compared to positive words. The latter were more often rated as self-relevant. Furthermore, there are least observations of allocations for neutral self-relevant words and positive non-self-relevant words. Negative allocations present the middle ground in both conditions, however, overall there appear to be more observations in the non-self-relevant negative and neutral allocations.

### Self-relevance and Valence, with the Influence of Intensity Ratings

**Fig. 2:**
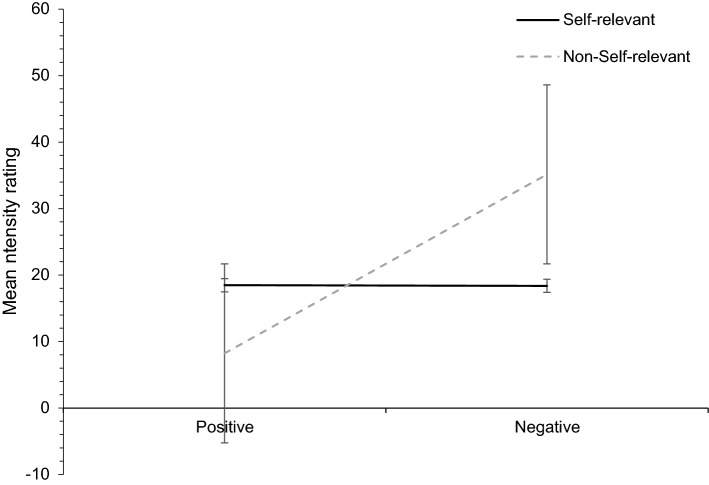
A depiction of the mean intensity ratings for positive and negative valenced information

Repeated-measures ANCOVA showed a significant main effect of valence (*F*(1, 53) = 5.210, *p* = 0.026, n_p_^2^ = 0.090) and a significant interaction effect of valence and self-relevance (*F*(1, 53) = 5.960, *p* = 0.018, n_p_^2^ = 0.101) on intensity. There was no main effect of self-relevance and no interaction effects of gender and age. Pairwise comparisons indicated a significant difference between positive and negative valence (*p* < 0.001) with a mean difference of 0.246 in favour of greater intensity for negative valence and an effect size of *d* = 0.53. For self-relevance, pairwise comparisons indicated no significant difference. Figure 2 displays the relationship between self-relevance, valence and intensity. The intensity for self-relevant allocations remained the same for positive and negative valence, however, intensity for non-self-relevant allocations varied. Negative non-self-relevant allocations garnered higher intensity as compared to negative self-relevant words. Contrastingly, positive self-relevant allocations indicated greater intensity ratings compared to positive non-self-relevant allocations. For positive valence self-relevant allocations resulted in greater intensity, while for negative valence, non-self-relevant obtained greater felt intensity ratings. Self-relevant neutral and self-relevant negative valence were equal in felt intensity. Overall, non-self-relevant negative allocations were the highest in felt intensity, while non-self-relevant positive allocations—the lowest.

### Word Analysis

The mode was calculated for each of the 552 words rated. Due to multi-allocations of words, the highest and second highest valid percent was shown (see Appendix D). There were 11 words (1.99%) which fell under more than one category (see Appendix E). Selection criteria indicated that for a multi-allocated word to be considered as representative of a valence and be placed within a word set, the valid percentage needed to exceed 50%. There were no instances of this, and therefore multi allocated words were not included in the finalised word sets. Overall, including multi allocations, positive valence acquired 108 words, neutral resulted in 196 words, and negative valence obtained 251 words. After applying the selection criteria, positive valence had 33 words, neutral valence—55 words, and negative valence had 205 words (see appendix F, G, H). After further removing words which failed the validity checks, the finalised negative set included 199 words, while the neutral set—48 words (see appendix I). Out of 552 words, 293 words (53.08%) were allocated as representing a valence with a high percentage of agreement between the participants, while 259 words (46.92%) were discarded due to low valid percentage and not suggested to be used in further research.

## Discussion

The present study aimed to analyse Dutch words with regard to valence, intensity, and self-relevance. The most important finding of the study is that self-relevance is valence dependent. Words rated as emotionally stimulating, negative and positive, had a greater proportion of self-relevance allocated to them, compared to neutral, which were most often non-self-relevant. Secondly, self-relevant words were not higher in felt intensity, unless valence was taken into consideration. There was no difference between intensity ratings of total self and non-self-relevant words, but there was a difference in intensity between negative and positive rated words. Negative valenced words obtained greater intensity ratings on non-self-relevant allocations, compared to self-relevant, while positive words indicated the opposite. Finally, the present findings suggest that valence, self-relevance and intensity ratings were gender and age independent.

The present findings confirm the hypothesis that the self is dependent on valence, and are in line with past research suggesting that valence is important in assigning self-relevance to words, such as Brown and Ure (1969). The findings also present evidence indicative of a positivity bias in healthy populations, with positive words being rated as more self-relevant compared to negative words. Negative words were more often rated as non-self-relevant. This phenomenon could be explained by the fact that individuals from the general population use positive words to describe themselves more often than they use negative words (Lopez et al., 2018). Past research has indicated that individuals who share positive information are evaluated more favourably (Forest & Wood, 2012). Therefore, to associate positive words as more relevant to the self is of interest to maintaining a social desirability status. Grühn and Smith (2008) provide further support to the strong relationship between valence and the self, in addition to indicating that positive words such as ‘tolerant’, ‘honest’, and ‘interested’ were rated as more self-relevant compared to negative words of the likes of ‘cruel’, ‘brutal’, and ‘dumb’, obtaining the lowest self-relevance allocations.

The finding that emotional valence is needed for a word to have self-relevance, is considered to be a novel and important finding, especially because past research has not addressed the self-relevance of neutral words. This can be explained by the fact that individuals are more likely to remember negative or positive information than neutral information and attribute a greater degree of relevance and meaning to it (Butler & James, 2010). The levels of processing theory (Craik & Lockhart, 1972) supports such occurrence by proposing that deeper levels of processing, being more effortful in both encoding and retrieval of information, will encourage a richer process of meaning attribution. Negative stimuli are shown to encourage more mental effort (Cacioppo et al., 1999), while positive stimuli evoke more internal, stable, and global attributions with regard to self-relevance (Mezulis et al., 2004), thus explaining the positivity effect evident within the self-relevance category. Based on such evidence, it is argued that emotionally charged stimuli require deeper levels of processing, and therefore, are linked to greater meaning attribution to the self and self-relevance (Bromgard et al., 2006).

The present findings also provide support for existing research (Benau et al., 2019; Bromgard et al., 2006) indicating that self-relevance influences greater levels of felt intensity, that is, within its representative valence. The current outcomes indicate that positively valenced self-relevant words have higher intensity compared to positive non-self-relevant words. This effect could be explained by the fact that words rated as self-relevant have, due to personal experience, a more intense meaning than words not related to the self. However, an interesting finding is that negatively valenced non-self-relevant words obtained greater intensity allocations than negative self-relevant counterparts. This is further supported by the present outcome that, while negatively valenced words are allocated a greater proportion of self-relevance, compared neutral words, they are also allocated a greater proportion of non-self-relevance, compared to positive words. An explanation of this is exhibited by the desire to maintain a positive, and therefore more attractive, self-image (Forest & Wood, 2012). The maintenance of a positive self-concept contributes to better mental wellbeing, while negative self-images have been associated with disorders such as social anxiety (Hulme et al., 2012). It has been found that compared to positive words, negative words elicit slower lexical decision, but faster valence judgements, indicating a selective responding on behalf of the individual, which is mediated by stimuli requiring faster response actions from individuals (Estes & Verges, 2008).

In contrast with findings from past research (Bellezza et al., 1986), the current findings disprove the hypothesis that valence, self-relevance, and intensity are gender dependent. Neither valence, self-relevance nor intensity ratings were affected by the gender of the participants. These two previous studies found systematic differences in pleasantness ratings between male and female raters, consequently, gender differences within the current study were also expected. However, compared to men, women did not use more extreme ratings when judging words on a pleasantness scale. Supporting this, Kinney et al. (2017) found no differences in attentional bias between men and women on negative and neutral information. Based on the present finding that the words were mostly negatively and neutrally valenced, together with the fact that it was negative and neutral words that the participants were asked to rate, it explains the lack of evident gender differences effects in the sample. Consequently, future studies should explore this connection further. Nonetheless, an advantage of the absence of gender differences is that the present word sets (see appendix I) can be used in studies including both male and female participants.

Moreover, age was not influential over valence, self-relevance or intensity ratings. To date there is little evidence about age-related differences in the perception and meaning of emotional material. Previous studies revealed an age effect on word valence evaluations (Grühn & Smith, 2008), when comparing the ratings of young (20–30 years of age) versus older adults (65–76 years of age). Older adults rated positive attributes as more positive, more arousing, and more relevant, while negative attributes were rated as less relevant than younger adults. Mezulis et al. (2004) also found that older adults display a positivity bias. The present findings cannot be deemed sufficient to state whether age influence results are in line with the positivity bias. Future research would benefit from exploring this issue, especially when younger adults are shown to be more distracted by, and able to recognise, negative words, while older adults attend equally to all stimuli, yet are shown to recognise positive words better (Thomas & Hasher, 2006).

Our study offers knowledge about the relationship between self-relevance and valence. This opens avenues for studying an individual’s self-definition, and can be useful for future psychological and psychiatric research purposes. Attentional biases towards negative information have been observed for many disorders, for example generalised anxiety disorder (GAD) (Kinney et al., 2017) and comorbid presentations of GAD and depression have shown an increase in the likelihood of looking for negative information in comparison to neutral (Mogg et al., 2000). Furthermore, Mogg and Bradley (2005) indicated that an attentional bias for negative information was found in depressed populations, but only when self-relevance was present. Nevertheless, an issue of the Kinney et al. (2017) and Mogg et al. (2000) studies is that they have all used facial recognition tasks. Evidence of word appraisals is still scarce, therefore, it is important to contribute to existing evidence base.

A limitation of the current study is the relatively small sample size of normal controls which undermines generalisation of the results. Another limitation is that only words previously labelled as neutral or negative were included, and positive stimuli were excluded. However, through ensuring a false positive option to the participants, truly neutral and negative valenced words could be discovered. The rating of emotional words can be highly dependent upon the context of other words presented (Gennari et al., 2007), so the rating option ‘positive’ was included in the design. As a result, a selection of negative and neutral words chosen from other studies was rated as positive in the current study, which indicates that emotional connotation is contaminated by the inclusion of positive words. Another reason for finding words to be rated as positive while they were initially included as neutral or negative on the basis of other reports is that these words were previously not validated as such. After validation, a part of the words was initially incorrectly categorized and actually are of positive valence. Therefore, it can be considered a strength that, even though a response bias cannot be ruled out, the study is not prone to an overestimation of negative valence of words due to inclusion of positive words during evaluation. On the contrary, the negative words are an underestimation for similar reasons. This makes the finding of the self and valence interaction even more robust. Another limitation is the degree of applicability to pathological samples, as the present sample was derived from the general population.

In conclusion, this study analysed 552 words with regard to valence intensity and self-relevance. Main outcomes indicated that valence is self-relevance dependent and that intensity of felt valence is self-relevance dependent. Gender and age differences were weak and future research is needed to confirm their status. Application of the findings in clinical populations needs to be further examined as the current study shows great potential to provide valuable insight into the relationship between the self and salience in these samples.

## Supplementary Information

Below is the link to the electronic supplementary material.

Supplementary file1 (DOCX 109 kb)

## References

[CR1] Agustí A, Satorres E, Pitarque A, Meléndez J (2017). An emotional Stroop task with faces and words. A comparison of young and older adults. Consciousness and Cognition.

[CR2] Badia A, Meneses J, Monereo C (2014). Affective dimension of university professors about their teaching: An exploration through the semantic differential technique. Universítas Psychologica.

[CR4] Bellezza F, Greenwald A, Banaji M (1986). Words high and low in pleasantness as rated by male and female college students. Behavior Research Methods Instruments & Computers.

[CR5] Benau E, Hill K, Atchley R, O’Hare A, Gibson L, Hajcak G, Ilardi SS, Foti D (2019). Increased neural sensitivity to self-relevant stimuli in major depressive disorder. Psychophysiology.

[CR6] Bradley, M. M. & Lang, P. J. (1999). *Affective norms for English words (ANEW): Stimuli, instruction manual and affective ratings, Technical Report C-1*. The Center for Research in Psychophysiology, University of Florida.

[CR7] Bradley MM, Lang PJ (2000). Affective reactions to acoustic stimuli. Psychophysiology.

[CR8] Bromgard G, Trafimow D, Bromgard I (2006). Valence of self-cognitions: The positivity of individual self-statements. The Journal of Social Psychology.

[CR9] Brosch T, Pourtois G, Sander D (2009). The perception and categorisation of emotional stimuli: A review. Cognition and Emotion.

[CR10] Brown WP, Ure DM (1969). Five rated characteristics of 650 word association stimuli. British Journal of Psychology.

[CR11] Burke PJ, Stets JE (2009). Identity theory.

[CR12] Butler AJ, James KH (2010). The neural correlates of attempting to suppress negative versus neutral memories. Cognitive Affective & Behavioral Neuroscience.

[CR13] Cacioppo J, Gardner W, Berntson G (1999). The affect system has parallel and integrative processing components: Form follows function. Journal of Personality and Social Psychology.

[CR14] Carroll J, Osgood C, Suci G, Tannenbaum P (1959). The Measurement of Meaning. Language.

[CR16] Cohen J (1988). Statistical power analysis for the behavioral sciences.

[CR17] Craik F, Lockhart R (1972). Levels of processing: A framework for memory research. Journal of Verbal Learning and Verbal Behavior.

[CR18] Crocetti E, Rubini M, Branje S, Koot H, Meeus W (2015). Self-concept clarity in adolescents and parents: A six-wave longitudinal and multi-informant study on development and intergenerational transmission. Journal of Personality.

[CR46] Estes Z, Verges M (2008). Freeze or flee? Negative stimuli elicit selective responding. Cognition.

[CR19] Fields, E., & Kuperberg, G. (2016). Dynamic effects of self-relevance and task on the neural processing of emotional words in context. *Frontiers In Psychology*, 6. 10.3389/fpsyg.2015.02003 PMC4710753 26793138

[CR20] Fischer A, Kret M, Broekens J (2018). Gender differences in emotion perception and self-reported emotional intelligence: A test of the emotion sensitivity hypothesis. PLoS ONE.

[CR21] Forest A, Wood J (2012). When social networking is not working. Psychological Science.

[CR22] Gennari SP, MacDonald MC, Postle BR, Seidenberg MS (2007). Context-dependent interpretation of words: Evidence for interactive neural processes. NeuroImage.

[CR23] Geraerts E, Smeets E, Jelicic M, van Heerden J, Merckelbach H (2005). Fantasy proneness, but not self-reported trauma is related to DRM performance of women reporting recovered memories of childhood sexual abuse. Consciousness and Cognition.

[CR24] Grühn D, Scheibe S (2008). Age-related differences in valence and arousal ratings of pictures from the International Affective Picture System (IAPS): Do ratings become more extreme with age?. Behavior Research Methods.

[CR25] Grühn D, Smith J (2008). Characteristics for 200 words rated by young and older adults: Age-dependent evaluations of German adjectives (AGE). Behavior Research Methods.

[CR26] Hermans D, De Houwer J (1994). Affective and subjective familiarity ratings of 740 Dutch words. Psychologica Belgica.

[CR27] Ho S, Mak C, Yeung D, Duan W, Tang S, Yeung J, Ching R (2015). Emotional valence, arousal, and threat ratings of 160 Chinese words among adolescents. PLoS ONE.

[CR28] Hoffmann H, Kessler H, Eppel T, Rukavina S, Traue H (2010). Expression intensity, gender and facial emotion recognition: Women recognize only subtle facial emotions better than men. Acta Psychologica.

[CR29] Hofmann M, Kuchinke L, Tamm S, Võ M, Jacobs A (2009). Affective processing within 1/10th of a second: High arousal is necessary for early facilitative processing of negative but not positive words. Cognitive, Affective, & Behavioral Neuroscience.

[CR30] Howell, D. C. (2010). *Statistical methods for psychology* (7th ed). Thomson Wadsworth.

[CR31] Hulme N, Hirsch C, Stopa L (2012). Images of the self and self-esteem: Do positive self-images improve self-esteem in social anxiety?. Cognitive Behaviour Therapy.

[CR32] Janschewitz K (2008). Taboo, emotionally valenced, and emotionally neutral word norms. Behavior Research Methods.

[CR33] Kinney K, Boffa J, Amir N (2017). Gender difference in attentional bias toward negative and positive stimuli in generalized anxiety disorder. Behavior Therapy.

[CR34] Lopez S, Pedrotti J, Snyder C (2018). Positive psychology: The scientific and practical explorations of human strengths.

[CR35] Mezulis A, Abramson L, Hyde J, Hankin B (2004). Is there a universal positivity bias in attributions? A meta-analytic review of individual, developmental, and cultural differences in the self-serving attributional bias. Psychological Bulletin.

[CR36] Mogg K, Bradley B (2005). Attentional bias in generalized anxiety disorder versus depressive disorder. Cognitive Therapy and Research.

[CR37] Mogg K, Millar N, Bradley B (2000). Biases in eye movements to threatening facial expressions in generalized anxiety disorder and depressive disorder. Journal of Abnormal Psychology.

[CR38] Osgood CE, Suci GJ, Tannenbaum PH (1957). The measurement of meaning.

[CR39] Sauer-Zavala S, Boswell J, Gallagher M, Bentley K, Ametaj A, Barlow D (2012). The role of negative affectivity and negative reactivity to emotions in predicting outcomes in the unified protocol for the transdiagnostic treatment of emotional disorders. Behaviour Research and Therapy.

[CR40] Ter Laak, J. (1992). *Emonet: A comparison of experimental measures of emotions with simulations by a network model* (Unpublished master's thesis). Unit of Theoretical and Experimental Psychology, Leiden University, The Netherlands.

[CR41] Thomaes K, Dorrepaal E, Draijer NP, de Ruiter MB, Elzinga BM, van Balkom AJ, Veltman DJ (2009). Increased activation of the left hippocampus region in Complex PTSD during encoding and recognition of emotional words: A pilot study. Psychiatry Research.

[CR42] Thomas R, Hasher L (2006). The influence of emotional valence on age differences in early processing and memory. Psychology and Aging.

[CR43] Tops M, van der Pompe G, Baas D, Mulder LJ, Den Boer JA, Meijman TF, Korf J (2003). Acute cortisol effects on immediate free recall and recognition of nouns depend on stimulus valence. Psychophysiology.

[CR44] Van Rensbergen B, De Deyne S, Storms G (2015). Estimating affective word covariates using word association data. Behavior Research Methods.

[CR45] Weiss H, Cropanzano R (1996). Affective Events Theory: A theoretical discussion of the structure, cause and consequences of affective experiences at work. Research in Organizational Behavior.

