# Supplementary material for: No Self Without Salience: Affective and Self-relevance Ratings of 552 Emotionally Valenced and Neutral Dutch Words
Source: J Psycholinguist Res. 2021 Jun 14;51(1):17–32. doi: 10.1007/s10936-021-09784-1 (PMC8930787; doi:10.1007/s10936-021-09784-1)
Supplement: Supplementary file 1 — Supplementary file1 (DOCX 109 kb) [file 10936_2021_9784_MOESM1_ESM.docx]

**SUPPLEMENTARY MATERIALS**

For paper entitled “No self without salience: affective and self-relevance ratings of 552 emotionally valenced and neutral Dutch words”

In submission for: Journal of Psycholinguistic Research

**Appendix A**.

All of the selected words with the translations of Dutch into English.

| **Dutch = English Translations** | | |
| --- | --- | --- |
| aandeel = share | aanklacht = charge | aanmaken = to prepare |
| aanslag = attack | aansporen = to urge | aantasten = to taint |
| aardbei = strawberry | abortus = abortion | absorptive = absorption |
| accent = accent | advocaat = lawyer | afblaffen = to bark at |
| afgaan = to fail | afgrijzen = horror | afgunst = envy |
| afhakken = to chop off | afkeer = aversion | afkeuring = disapproval |
| afkraken = to decry | afnemen = to take away | afscheid = goodbye |
| afschuw = revulsion | afsnauwen = to snap at | aftrekken = to masturbate or to deduct |
| afval = trash | afwachten = to wait and see | afwijzing = rejection |
| agressie = aggression | alcohol = alcohol | ambtenaar = civil servant |
| angst = fear | anker = anchor | antwoord = answer |
| appel = apple | appelflap = apple turnover | arbeid = labor |
| archief = archive | architect = architect | argwaan = suspicion |
| armoede = poverty | asbak = ashtray | atoombom = nuclear bomb |
| baard = beard | badkamer = bathroom | bakken = baking or to bake |
| balkon = balcony | balpen = ballpoint pen | banaan = banana |
| basketbal = basketball | bedreigen = to threaten | bedrieger = deceiver |
| bedrijf = company | bedrog = deceit | beklemmen = to oppress |
| belazerd = fooled | beleggen = to invest | beleid = policy |
| bellen = to call | benauwen = to agitate | beroerd = miserable |
| beroerte = stroke | besmetten = to contaminate | bespotten = to mock |
| bespreken = to discuss | bestaan = to exist or existence | bestek = cutlery |
| bestuur = board | betasten = to touch | beven = to shiver |
| biljet = note | blaam = blame | bladzijde = page |
| blikje = can | blinddoek = blindfold | bloed = blood |
| bloedbad = bloodbath | bloeden = bleeding or to bleed | blunder = blunder |
| boete = fine | bordeel = brothel | borduren = to embroider |
| borsten = breasts | boter = butter | braaksel = vomit |
| broek = pants | brommer = moped | buikpijn = stomach ache |
| bureau = desk | burger = citizen | chanteren = to blackmail |
| chauffeur = driver | cirkel = circle | citaat = quote |
| conclusive = conclusion | condom = condom | conflict = conflict |
| consulaat = consulate | contact = contact | crimineel = criminal |
| crisis = crisis | dader = perpetrator | dagblad = newspaper |
| dakgoot = gutter | depressive = depression | deurknop = door knob |
| dialect = dialect | diarree = diarrhoea | diefstal = theft |
| dineren = to dine | diploma = diploma | disco = disco |
| document = document | dodelijk = deadly | doden = to kill |
| doodgaan = to die | doodslag = manslaughter | doodsteek = deathblow |
| doodstraf = death penalty | dossier = file | douche = shower |
| dozijn = dozen | drama = drama | drammen = to urge or to whine |
| dreigen = to threaten | dreun = thump | droefheid = sadness |
| droevig = sad | drugs = drugs | dubbel = double |
| duister = dark | dwang = coercion or force | dwerg = dwarf |
| dwingen = to coerce or to force | eetlepel = tablespoon | eicel = ovum |
| element = element | elleboog = elbow | ellende = misery |
| erectie = erection | ergeren = to annoy | ergernis = annoyance |
| erotisch = erotic | etiket = label | etter = pus |
| faculteit = faculty | fallen = to fail | fiets = bike |
| fietsbel = bike bell | filmen = to film | firma = firm |
| flacon = bottle or vial | flirten = to flirt | folteren = to torture |
| fornuis = stove or furnace | functie = function | ganzenbord = game of the goose |
| gebak = pastry | gebouw = building | gebrek = lack |
| geheim = secret | gehoor = hearing | geluid = sound |
| getreiter = harassment | gevaar = danger | gevecht = fight |
| geweld = violence | geweten = conscience | gezicht = face |
| gezwel = tumor | gifslang = venomous snake | gijzeling = kidnapping |
| gitaar = guitar | glaasje = glass | gordijn = curtain |
| graan = grain | griep = flu | grijs = gray |
| groen = green | groente = vegetable | groep = group |
| grondstof = natural resource | haarbos = head of hair | hagedis = lizard |
| haken = hooks or to hook | hakmes = chopping knife | handel = trade |
| handtas = handbag | haten = to hate | hebben = to have |
| heelal = universe | hekel = aversion | hertogin = duchess |
| heuvel = hill | honger = hunger | honing = honey |
| hopeloos = hopeless | horloge = watch | houding = posture |
| huilen = to cry | huiveren = to shudder | hypocrite = hypocrite |
| hysterie = hysteria | ijzer = iron | inbreak = burglary |
| incest = incest | infectie = infection | injectie = injection |
| instituut = institute | instorten = collapse | ivoor = ivory |
| jaloezie = jealousy | janken = to whimper | jarretel = suspender |
| jennen = to dog or to tease | cabinet = cabinet | cadaver = cadaver |
| kader = framework | kanaal = canal | kanker = cancer |
| kantoor = office | kapper = hairdresser | katten = cats |
| kelder = basement | kenteken = license plate | kerkhof = graveyard |
| kermen = to groan | kever = beetle | klacht = complaint |
| klant = customer | klasse = class | knorrig = grumpy |
| koelkast = fridge | koffie = coffee | kogel = bullet |
| kokhalzen = retching | komen = to come | koorts = fever |
| korting = discount | kostuum = costume | kotsen = to vomit |
| kozijn = window frame | kraan = tap | krant = newspaper |
| krenken = to hurt | krijsen = to scream | kritiek = critique |
| kruid = herb | kussen = to kiss | kutje = pussy |
| kwaal = ailment | kwellen = to torment | kwetsen = to hurt |
| laden = to load | lafaard = coward | laken = bed sheet |
| landbouw = agriculture | laster = slander | lasting = difficult |
| lawaai = noise | legpuzzel = jigsaw puzzle | lenen = to borrow or to loan |
| lepra = leprosy | leraar = teacher | letsel = injury |
| leugen = lie | leuning = railing | levenloos = lifeless |
| liegen = to lie | lijden = to suffer | links = left |
| lopen = to walk | lusteloos = listless | magazijn = warehouse |
| maken = to make | martelen = to torture | matras = mattress |
| meedelen = to inform | meeloper = opportunist | meester = master |
| mening = opinion | messteek = knife stab | metaal = metal |
| meten = measure | methode = method | metselaar = bricklayer |
| meubel = furniture | middle = middle | minnaar = lover |
| misdaad = crime | misdrijf = crime | miskraam = miscarriage |
| misleiden = to deceive | mislukt = failed | mismaakt = deformed |
| misvormen = to deform | moedeloos = despondent | moeten = to must |
| monster = monster | moord = murder | naald = needle |
| naspel = aftermath | nederlaag = defeat | neuken = to fuck |
| noodkreet = cry for help | noodlot = fate | octaaf = octave |
| oever = shore | omelette = omelette | omkomen = to perish |
| omroep = broadcasting | onderzoek = research | ongeluk = accident |
| ongeval = accident | onkruid = weeds | onlust = disturbance |
| onmacht = powerlessness | onrecht = injustice | ontbijt = breakfast |
| ontrouw = unfaithful | ontslag = resignation or dismissal | onzeker = uncertain |
| oorlog = war | ophangen = to hang | oplichten = to scam |
| opsluiten = to lock up | opwinden = to turn on or to excite | orgasme = orgasm |
| orgie = orgy | overleg = consult | paniek = panic |
| papier = paper | parade = parade | paragraaf = paragraph |
| paring = mating or pair | passen = to fit | pedofiel = paedophile |
| piano = piano | piekeren = to mull | pijpen = piper or to give a blowjob |
| pilaar = pillar | pinda = peanut | plafond = ceiling |
| plank = plank | politie = police | portret = portrait |
| postzegel = stamp | potlood = pencil | priester = priest |
| prijs = prize or price | problem = problem | profiteur = freeloader |
| programma = program | project = project | provincie = province |
| pudding = pudding | raadsel = riddle | razernij = fury |
| reden = reason | regel = rule | regenton = rain barrel |
| register = register | reizen = to travel | rendement = returns |
| rente = interest | reuma = rheumatism | rijmen = to rhyme |
| rillen = to shiver | rivier = river | roddel = gossip |
| roeren = to stir | roofmoord = robbery with murder | rouwen = to mourn |
| ruzie = fight or quarrel | sadist = sadist | sandaal = sandal |
| sarren = to dog | schaamte = embarrassment | schaar = scissors |
| schaden = to damage | schandaal = scandal | schande = disgrace |
| scheiding = separation | schelden = to curse | schoen = shoe |
| schoft = bastard | schok = shock | school = school |
| schreeuw = scream | schroef = screw | schuld = debt |
| schuren = to polish | schurft = scabies | seksfilm = sex movie |
| september = september | sigaar = cigar | sigaret = cigarette |
| slaan = to hit | slaapzaal = dormitory | slager = butcher |
| sleutel = key | slijmen = suck up to | slipje = panties |
| sloot = ditch | smoking = tuxedo | snauwen = to snarl |
| sneuvelen = to die | sperma = sperm | spijten = to regret |
| spinazie = spinach | spoelen = to flush or to rinse | sport = sport |
| stadhuis = city hall | stank = stench | steekwond = stab wound |
| steelpan = saucepan | steen = stone | steil = steep |
| stellen = to set | sterven = to die | stier = bull |
| stikken = to suffocate | stoel = chair | stoelpoot = chair leg |
| stoep = sidewalk | stomerij = drycleaner | straat = street |
| straf = punishment | straffen = to punish | straling = radiation |
| stranden = to strand | stukadoor = plasterer | tart = cake |
| tafel = table | taken = branches | tapijt = tapestry |
| tarten = to defy | tarwe = wheat | tegel = tile |
| tegenslag = setback | tegenzin = reluctance | teisteren = to plague |
| tekenen = to draw | tekort = shortage | telefoon = telephone |
| telegram = telegram | teller = counter | termijn = tea towel |
| theedoek = tea towel | theelepel = teaspoon | theorie = theory |
| tijdperk = era | timmerman = carpenter | tiran = tyrant |
| tobben = to worry | toetje = dessert | toilet = toilet |
| tongzoen = french kiss | traditie = route | traject = route |
| trauma = trauma | trede = step | treden = to step |
| treiteren = to harass | trekken = to pull | treuren = to be sad |
| triest = sad | trompet = trumpet | troosten = to comfort |
| trottoir = pavement | twijfelen = to doubt | uitgever = publisher |
| uitjouwen = to boo | uitlachen = to laugh at | uitzenden = to send out |
| urine = urine | vagina = vagina | vakantie = holiday |
| varen = to sail | venster = window | verband = connection |
| verdord = withered | verdriet = sadness | verdwalen = to get lost |
| vergelijk = compare | vergroten = enlarge | verlammen = to paralyze |
| verlies = loss | verlinken = to betray | verloren = lost |
| vernielen = to vandalize | verraad = betrayal | versie = version |
| vertalen = to translate | verveling = boredom | vervoer = transport |
| verwijten = to blame | verzuipen = to drown | vierkant = square |
| vijand = enemy | vingers = fingers | vlees = meat |
| vleugel = wing | vliegtuig = airplane | vloer = floor |
| vluchten = to flee | vodka = vodka | voertuig = vehicle |
| voetbal = football | voetpad = footpath | voorstel = proposal |
| vrees = fear | vrijen = to make love | waanzin = insanity |
| walging = disgust | wandelen = to walk | wanhoop = despair |
| wanhopen = to despair | wapen = weapon | water = water |
| weemoed = melancholy | weigering = refusal | wereld = world |
| werkeloos = unemployed | werken = to work | winkel = shop |
| winkelier = retailer | winter = winter | wippen = to seesaw or to have sex |
| woede = anger | woedend = angry | wonen = to live |
| word = word | wormen = worms | wortel = carrot or root |
| wraak = revenge | wreedheid = cruelty | wurgen = to strangle |
| zakje = little bag | zandloper = hourglass | zegel = seal |
| zeiken = to nag or to pee | zelfmoord = suicide | zetel = seat |
| zeuren = to nag | ziekte = illness | zielig = pitiful |
| zitplaats = seat | zoenen = to kiss | zondebok = scapegoat |
| zonlicht = sunlight | zuchten = to sigh | zwakheid = weakness |

*Note*: *N* = 552 words

**Appendix B**.

Words excluded from Cronbach’s alpha analysis due to missing values.

| **Words excluded from scales** |
| --- |
| **Valence**  afblaffen, afkraken, bedrieger, bedrog, diefstal, ellende, gijzeling, incest, kwetsen, martelen, moedeloos, onmacht, onrecht, paniek, pedofiel, roofmoord, treiteren, verraad, zeuren, zoenen |
| **Self-relevance**  absorptie, dozijn, hertogin, telegram |

*Note*: intensity not included in analysis as neutral valence was not allocated an intensity score, resulting in high number of missing values.

**Appendix C.**

Validation of words and participants.

|  | **Coefficient of variation fail** | **>70% missing cases** | **Failed participants** |
| --- | --- | --- | --- |
| **Validity scale** | afblaffen; bedrieger; bedrog; diefstal; ellende; zeuren | - | Participant 8 |
| **Intensity scale** | absorptie; dozijn; dubbel; metaal; plafond; stomerij; trede; zetel | aandeel; absorptie; afnemen; archief; balpen; bedrijf; beleid; bestek; bladzijde; blikje; blinddoek; bureau; burger; chauffeur; cirkel; citaat; consulaat; dakgoot; deurknop; document; dossier; dozijn; dubbel; eetlepel; elleboog; etiket; firma; flacon; gebouw; grondstof; hertogin; houding; ijzer; injectie; kabinet; kader; kanaal; kenteken; kozijn; kraan; laden; leuning; links; magazijn; meedelen; metaal; meten; methode; middel; octaaf; omroep; paragraaf; pilaar; plafond; plank; postzegel; potlood; reden; register; roeren; schaar; schroef; sloot; spoelen; steelpan; steen; steil; stellen; stoel; stoelpoot; stoep; stomerij; straat; takken; tegel; telegram; teller; theedoek; theelepel; tijdperk; traject; trede; treden; trekken; trottoir; uitgever; urine; vergelijk; versie; vierkant; vloer; woord; zakje; zandloper; zegel; zetel | Participant 8  Participant 51 |
| **Self-relevance scale** | dozijin; hertogin | - | Participant 8 |

*Note*: total words in the analysis N = 552.

**Appendix D**.

All allocated words to corresponding valence options.

| **Word** | **Associated valence** | | | | |
| --- | --- | --- | --- | --- | --- |
|  | **Positive** | **Neutral** | **Negative** | **Both positive and negative** | **Cannot decide** |
| aandeel |  | √ (30, 56.6%) |  |  |  |
| aanklacht |  |  | √ (36, 66.7%) |  |  |
| aanmaken |  | √ (35, 64.8%) |  |  |  |
| aanslag |  |  | √ (53, 96.4%) |  |  |
| aansporen | √ (33, 62.3%) |  |  |  |  |
| aantasten |  |  | √ (40, 75.5%) |  |  |
| aardbei | √ (51, 94.4%) |  |  |  |  |
| abortus |  |  | √ (23, 41.8%) |  |  |
| absorptie |  | √ (43, 79.6%) |  |  |  |
| accent |  | √ (17, 30.9%) |  |  |  |
| advocaat |  | √ (25, 46.3%) |  |  |  |
| afblaffen |  |  | √ (53, 100%) |  |  |
| afgaan |  |  | √ (41, 77.4%) |  |  |
| afgrijzen |  |  | √ (49, 89.1%) |  |  |
| afgunst |  |  | √ (50, 92.6%) |  |  |
| afhakken |  |  | √ (43, 78.2%) |  |  |
| afkeer |  |  | √ (49, 90.7%) |  |  |
| afkeuring |  |  | √ (47, 85.5%) |  |  |
| afkraken |  |  | √ (53, 98.1%) |  |  |
| afnemen |  | √ (20, 37%) |  |  |  |
| afscheid |  |  | √ (31, 56.4%) |  |  |
| afschuw |  |  | √ (50, 94%) |  |  |
| afsnauwen |  |  | √ (53, 98.1%) |  |  |
| aftrekken |  | √ (25; 44.6%) |  |  |  |
| afval |  |  | √ (28, 52.8%) |  |  |
| **afwachten*** |  | √ (16, 29.6%) | √ (16, 29.6%) | √ (21, 38.9%) |  |
| afwijzing |  |  | √ (52, 98.2%) |  |  |
| agressie |  |  | √ (52, 94.5%) |  |  |
| **alcohol*** |  |  | √ (13, 23.6%) | √ (18, 32.7%) |  |
| ambtenaar |  | √ (22, 40%) |  |  |  |
| angst |  |  | √ (46, 85.2%) |  |  |
| anker | √ (28, 50.9%) |  |  |  |  |
| antwoord |  | √ (21, 39.6%) |  |  |  |
| appel | √ (35, 64.8%) |  |  |  |  |
| appelflap | √ (41, 74.5%) |  |  |  |  |
| arbeid | √ (29, 55.8%) |  |  |  |  |
| archief |  | √ (35, 64.8%) |  |  |  |
| architect | √ (26, 47.3%) |  |  |  |  |
| argwaan |  |  | √ (47, 87%) |  |  |
| armoede |  |  | √ (51, 92.7%) |  |  |
| asbak |  |  | √ (35, 64.8%) |  |  |
| atoombom |  |  | √ (51, 92.7%) |  |  |
| baard |  | √ (27, 51.9%) |  |  |  |
| badkamer | √ (35, 66%) |  |  |  |  |
| bakken | √ (38, 70.4%) |  |  |  |  |
| balkon |  | √ (26, 49.1%) |  |  |  |
| balpen |  | √ (41, 74.5%) |  |  |  |
| banaan | √ (29, 53.7%) |  |  |  |  |
| basketbal |  | √ (29, 53.7%) |  |  |  |
| bedreigen |  |  | √ (52, 98.1%) |  |  |
| bedrieger |  |  | √ (54, 100%) |  |  |
| bedrijf |  | √ (29, 54.7%) |  |  |  |
| bedrog |  |  | √ (54, 100%) |  |  |
| beklemmen |  |  | √ (52, 94.5%) |  |  |
| belazerd |  |  | √ (52, 96.3%) |  |  |
| beleggen |  | √ (26, 48.1%) |  |  |  |
| beleid |  | √ (25, 46.3%) |  |  |  |
| bellen | √ (25, 45.5%) |  |  |  |  |
| benauwen |  |  | √ (52, 96.3%) |  |  |
| beroerd |  |  | √ (51, 94.4%) |  |  |
| beroerte |  |  | √ (52, 96.3%) |  |  |
| besmetten |  |  | √ (48, 88.9%) |  |  |
| bespotten |  |  | √ (48, 92.3%) |  |  |
| bespreken | √ (23, 42.6%) |  |  |  |  |
| bestaan | √ (32, 58.2%) |  |  |  |  |
| bestek |  | √ (42, 79.2%) |  |  |  |
| bestuur |  | √ (28, 51.9%) |  |  |  |
| betasten |  |  | √ (21, 38.9%) |  |  |
| beven |  |  | √ (40, 74.1%) |  |  |
| biljet |  | √ (31, 57.4%) |  |  |  |
| blaam |  |  | √ (31, 57.4%) |  |  |
| bladzijde |  | √ (43, 79.6%) |  |  |  |
| blikje |  | √ (41, 74.5%) |  |  |  |
| blinddoek |  | √ (26, 49.1%) |  |  |  |
| bloed |  |  | √ (22, 41.5%) |  |  |
| bloedbad |  |  | √ (50, 90.9%) |  |  |
| bloeden |  |  | √ (40, 76.9%) |  |  |
| blunder |  |  | √ (40, 74.1%) |  |  |
| boete |  |  | √ (45, 83.3%) |  |  |
| bordeel |  |  | √ (23, 42.6%) |  |  |
| borduren |  | √ (28, 50.9%) |  |  |  |
| borsten | √ (36, 66.7%) |  |  |  |  |
| boter |  | √ (29, 55.8%) |  |  |  |
| braaksel |  |  | √ (50, 92.6%) |  |  |
| broek |  | √ (33, 61.1%) |  |  |  |
| brommer |  | √ (25, 45.5%) |  |  |  |
| buikpijn |  |  | √ (45, 86.5%) |  |  |
| bureau |  | √ (39, 72.2%) |  |  |  |
| burger |  | √ (36, 67.9%) |  |  |  |
| chanteren |  |  | √ (52, 98.1%) |  |  |
| chauffeur |  | √ (40, 75.5%) |  |  |  |
| cirkel |  | √ (35, 64.8%) |  |  |  |
| citaat |  | √ (42, 79.2%) |  |  |  |
| conclusie |  | √ (23, 42.6%) |  |  |  |
| condoom | √ (22, 40%) |  |  |  |  |
| conflict |  |  | √ (42, 80.8%) |  |  |
| consulaat |  | √ (38, 70.4%) |  |  |  |
| contact | √ (42, 77.8%) |  |  |  |  |
| crimineel |  |  | √ (50, 92.6%) |  |  |
| crisis |  |  | √ (38, 70.4%) |  |  |
| dader |  |  | √ (40, 75.5%) |  |  |
| **dagblad*** | √ (25, 46.6%) | √ (25, 46.6%) |  |  |  |
| dakgoot |  | √ (43, 81.1%) |  |  |  |
| depressie |  |  | √ (52, 94.5%) |  |  |
| deurknop |  | √ (51, 94.4%) |  |  |  |
| dialect | √ (20, 37%) |  |  |  |  |
| diarree |  |  | √ (50, 92.6%) |  |  |
| diefstal |  |  | √ (54, 100%) |  |  |
| dineren | √ (46, 85.2%) |  |  |  |  |
| diploma | √ (40, 76.9%) |  |  |  |  |
| disco | √ (33, 60%) |  |  |  |  |
| document |  | √ (38, 71.7%) |  |  |  |
| dodelijk |  |  | √ (44, 80%) |  |  |
| doden |  |  | √ (48, 90.6%) |  |  |
| doodgaan |  |  | √ (40, 75.5%) |  |  |
| doodslag |  |  | √ (52, 96.3%) |  |  |
| doodsteek |  |  | √ (52, 96.3%) |  |  |
| doodstraf |  |  | √ (39, 72.2%) |  |  |
| dossier |  | √ (34, 64.2%) |  |  |  |
| douche | √ (46, 88.5%) |  |  |  |  |
| dozijn |  | √ (46, 86.8%) |  |  |  |
| drama |  |  | √ (27, 49.1%) |  |  |
| drammen |  |  | √ (50, 90.9%) |  |  |
| dreigen |  |  | √ (52, 94.5%) |  |  |
| dreun |  |  | √ (45, 80.4%) |  |  |
| droefheid |  |  | √ (46, 85.2%) |  |  |
| droevig |  |  | √ (45, 83.3%) |  |  |
| drugs |  |  | √ (40, 74.1%) |  |  |
| dubbel |  | √ (35, 64.8%) |  |  |  |
| duister |  |  | √ (21, 39.6%) |  |  |
| dwang |  |  | √ (52, 94.5%) |  |  |
| dwerg |  | √ (27, 48.2%) |  |  |  |
| dwingen |  |  | √ (51, 94.4%) |  |  |
| eetlepel |  | √ (39, 70.9%) |  |  |  |
| eicel |  | √ (27, 50%) |  |  |  |
| element |  | √ (32, 59.3%) |  |  |  |
| elleboog |  | √ (40, 75.5%) |  |  |  |
| ellende |  |  | √ (53, 100%) |  |  |
| erectie | √ (37, 68.5%) |  |  |  |  |
| ergeren |  |  | √ (53, 98.1%) |  |  |
| ergernis |  |  | √ (54, 98.2%) |  |  |
| erotisch | √ (41, 74.5%) |  |  |  |  |
| etiket |  | √ (33, 62.3%) |  |  |  |
| etter |  |  | √ (46, 83.6%) |  |  |
| faculteit |  | √ (30, 55.6%) |  |  |  |
| falen |  |  | √ (50, 90.9%) |  |  |
| fiets | √ (39, 73.6%) |  |  |  |  |
| fietsbel |  | √ (35, 64.8%) |  |  |  |
| filmen | √ (28, 52.8%) |  |  |  |  |
| firma |  | √ (38, 70.4%) |  |  |  |
| flacon |  | √ (43, 79.6%) |  |  |  |
| flirten | √ (41, 75.9%) |  |  |  |  |
| folteren |  |  | √ (50, 94.3%) |  |  |
| fornuis |  | √ (28, 52.8%) |  |  |  |
| functie |  | √ (30, 55.6%) |  |  |  |
| ganzenbord | √ (29, 51.8%) |  |  |  |  |
| gebak | √ (41, 77.4%) |  |  |  |  |
| gebouw |  | √ (39, 72.2%) |  |  |  |
| gebrek |  |  | √ (45, 84.9%) |  |  |
| geheim* |  |  | √ (19, 35.2%) | √ (21, 38.9%) |  |
| gehoor | √ (26, 48.1%) |  |  |  |  |
| geluid |  | √ (19, 34.5%) |  |  |  |
| getreiter |  |  | √ (52, 94.5%) |  |  |
| gevaar |  |  | √ (47, 87%) |  |  |
| gevecht |  |  | √ (43, 79.6%) |  |  |
| geweld |  |  | √ (51, 94.4%) |  |  |
| geweten | √ (21, 37.5%) |  |  |  |  |
| gezicht | √ (31, 57.4%) |  |  |  |  |
| gezwel |  |  | √ (50, 96.2%) |  |  |
| gifslang |  |  | √ (38, 69.1%) |  |  |
| gijzeling |  |  | √ (52, 96.3%) |  |  |
| gitaar | √ (36, 65.5%) |  |  |  |  |
| glaasje |  | √ (27, 50.9%) |  |  |  |
| gordijn |  | √ (33, 63.5%) |  |  |  |
| graan | √ (28, 50%) |  |  |  |  |
| griep |  |  | √ (42, 76.4%) |  |  |
| grijs |  | √ (24, 44.4%) |  |  |  |
| groen | √ (42, 75%) |  |  |  |  |
| groente | √ (42, 79.2%) |  |  |  |  |
| groep |  | √ (21, 38.9%) |  |  |  |
| grondstof |  | √ 37, 68.5%) |  |  |  |
| haarbos |  | √ (26, 48.1%) |  |  |  |
| hagedis |  | √ (37, 67.3%) |  |  |  |
| haken |  | √ (35, 64.8%) |  |  |  |
| hakmes |  |  | √ (26, 48.1%) |  |  |
| handel |  | √ (25, 45.5%) |  |  |  |
| handtas |  | √ (32, 59.3%) |  |  |  |
| haten |  |  | √ (49, 94.2%) |  |  |
| hebben |  | √ (21, 38.2%) |  |  |  |
| heelal | √ (35, 63.6%) |  |  |  |  |
| hekel |  |  | √ (41, 77.4%) |  |  |
| hertogin |  | √ (35, 66%) |  |  |  |
| heuvel |  | √ (31, 58.5%) |  |  |  |
| honger |  |  | √ (39, 72.2%) |  |  |
| honing | √ (39, 73.6%) |  |  |  |  |
| hopeloos |  |  | √ (49, 90.7%) |  |  |
| horloge | √ (30, 53.6%) |  |  |  |  |
| **houding*** |  | √ (19, 35.2%) |  | √ (22, 40.7%) |  |
| **huilen*** |  |  | √ (16, 30.2%) | √ (26, 49.1%) |  |
| huiveren |  |  | √ (38, 70.4%) |  |  |
| hypocriet |  |  | √ (50, 90.9%) |  |  |
| hysterie |  |  | √ (43, 78.2%) |  |  |
| ijzer |  | √ (38, 70.4%) |  |  |  |
| inbraak |  |  | √ (52, 96.3%) |  |  |
| incest |  |  | √ (54, 98.2%) |  |  |
| infectie |  |  | √ (46, 85.2%) |  |  |
| injectie |  | √ (19, 35.2%) |  |  |  |
| instituut |  | √ (26, 48.1%) |  |  |  |
| instorten |  |  | √ (49, 90.7%) |  |  |
| ivoor |  | √ (23, 42.6%) |  |  |  |
| jaloezie |  |  | √ (45, 81.8%) |  |  |
| janken |  |  | √ (35, 64.8%) |  |  |
| jarretel |  | √ (28, 53.8%) |  |  |  |
| jennen |  |  | √ (43, 81.1%) |  |  |
| kabinet |  | √ (23, 42.6%) |  |  |  |
| kadaver |  |  | √ (38, 69.1%) |  |  |
| kader |  | √ (38, 71.7%) |  |  |  |
| kanaal |  | √ (36, 67.9%) |  |  |  |
| kanker |  |  | √ (53, 96.4%) |  |  |
| kantoor |  | √ (31, 59.6%) |  |  |  |
| kapper | √ (34, 60.7%) |  |  |  |  |
| katten | √ (22, 42.3%) |  |  |  |  |
| kelder |  | √ (29, 54.7%) |  |  |  |
| kenteken |  | √ (39, 72.2%) |  |  |  |
| kerkhof |  |  | √ (23, 41.8%) |  |  |
| kermen |  |  | √ (44, 81.5%) |  |  |
| kever |  | √ (25, 47.2%) |  |  |  |
| klacht |  |  | √ (40, 71.4%) |  |  |
| klant |  | √ (27, 49.1%) |  |  |  |
| klasse | √ (22, 39.3%) |  |  |  |  |
| knorrig |  |  | √ (36, 66.7%) |  |  |
| koelkast |  | √ (30, 55.6%) |  |  |  |
| koffie | √ (37, 68.5%) |  |  |  |  |
| kogel |  |  | √ (42, 79.2%) |  |  |
| kokhalzen |  |  | √ (48, 90.6%) |  |  |
| komen |  | √ (25, 47.2%) |  |  |  |
| koorts |  |  | √ (46, 83.6%) |  |  |
| korting | √ (46, 82.1%) |  |  |  |  |
| kostuum | √ (27, 49.1%) |  |  |  |  |
| kotsen |  |  | √ (49, 90.7%) |  |  |
| kozijn |  | √ (47, 87%) |  |  |  |
| kraan |  | √ (38, 70.4%) |  |  |  |
| krant | √ (33, 62.3%) |  |  |  |  |
| krenken |  |  | √ (50, 90.9%) |  |  |
| krijsen |  |  | √ (46, 86.8%) |  |  |
| **kritiek*** |  |  | √ (23, 42.6%) | √ (23, 42.6%) |  |
| kruid | √ (26, 49.1%) |  |  |  |  |
| kussen | √ (47, 88.7%) |  |  |  |  |
| kutje |  |  | √ (23, 41.8%) |  |  |
| kwaal |  |  | √ (48, 88.9%) |  |  |
| kwellen |  |  | √ (52, 96.3%) |  |  |
| kwetsen |  |  | √ (51, 98.1%) |  |  |
| laden |  | √ (36, 65.5%) |  |  |  |
| lafaard |  |  | √ (50, 92.6%) |  |  |
| laken |  | √ (31, 55.4%) |  |  |  |
| landbouw | √ (26, 47.3%) |  |  |  |  |
| laster |  |  | √ (48, 85.7%) |  |  |
| lastig |  |  | √ (32, 58.2%) |  |  |
| lawaai |  |  | √ (44, 81.5%) |  |  |
| legpuzzel |  | √ (31, 59.6%) |  |  |  |
| **lenen*** |  |  | √ (18, 33.3%) | √ (20, 37%) |  |
| lepra |  |  | √ (46, 83.6%) |  |  |
| leraar | √ (22, 40.7%) |  |  |  |  |
| letsel |  |  | √ (45, 83.3%) |  |  |
| leugen |  |  | √ (47, 87%) |  |  |
| leuning |  | √ (40, 71.4%) |  |  |  |
| levenloos |  |  | √ (46, 83.6%) |  |  |
| liegen |  |  | √ (48, 92.3%) |  |  |
| lijden |  |  | √ (50, 96.2%) |  |  |
| links |  | √ (30, 56.6%) |  |  |  |
| lopen | √ (32, 60.4%) |  |  |  |  |
| lusteloos |  |  | √ (48, 88.9%) |  |  |
| magazijn |  | √ (44, 81.5%) |  |  |  |
| maken | √ (34, 61.8%) |  |  |  |  |
| martelen |  |  | √ (53, 98.1%) |  |  |
| matras | √ (28, 53.8%) |  |  |  |  |
| meedelen |  | √ (29, 53.7%) |  |  |  |
| meeloper |  |  | √ (47, 88.7%) |  |  |
| meester |  | √ (23, 43.4%) |  |  |  |
| mening | √ (26, 47.3%) |  |  |  |  |
| messteek |  |  | √ (49, 92.5%) |  |  |
| metaal |  | √ (45, 81.8%) |  |  |  |
| meten |  | √ (34, 63%) |  |  |  |
| methode |  | √ (31, 57.4%) |  |  |  |
| metselaar |  | √ (31, 56.4%) |  |  |  |
| meubel |  | √ (34, 66.7%) |  |  |  |
| middel |  | √ (41, 75.9%) |  |  |  |
| minnaar | √ (21, 38.9%) |  |  |  |  |
| misdaad |  |  | √ (49, 92.5%) |  |  |
| misdrijf |  |  | √ (51, 94.4%) |  |  |
| miskraam |  |  | √ (48, 87.3%) |  |  |
| misleiden |  |  | √ (50, 90.9%) |  |  |
| mislukt |  |  | √ (50, 92.6%) |  |  |
| mismaakt |  |  | √ (50, 94.3%) |  |  |
| misvormen |  |  | √ (47, 87%) |  |  |
| moedeloos |  |  | √ (52, 96.3%) |  |  |
| moeten |  |  | √ (38, 69.1%) |  |  |
| monster |  |  | √ (25, 46.3%) |  |  |
| moord |  |  | √ (53, 94.6%) |  |  |
| naald |  | √ (26, 48.1%) |  |  |  |
| naspel | √ (33, 62.3%) |  |  |  |  |
| nederlaag |  |  | √ (44, 81.5%) |  |  |
| neuken | √ (19, 35.2%) |  |  |  |  |
| noodkreet |  |  | √ (49, 90.7%) |  |  |
| noodlot |  |  | √ (40, 74.1%) |  |  |
| octaaf |  | √ (38, 69.1%) |  |  |  |
| **oever*** | √ (25, 48.1%) | √ (25, 48.1%) |  |  |  |
| omelet | √ (33, 61.1%) |  |  |  |  |
| omkomen |  |  | √ (49, 89.1%) |  |  |
| omroep |  | √ (38, 69.1%) |  |  |  |
| onderzoek | √ (19, 34.5%) |  |  |  |  |
| ongeluk |  |  | √ (53, 98.1%) |  |  |
| ongeval |  |  | √ (51, 94.4%) |  |  |
| onkruid |  |  | √ (27, 50%) |  |  |
| onlust |  |  | √ (40, 71.4%) |  |  |
| onmacht |  |  | √ (53, 98.1%) |  |  |
| onrecht |  |  | √ (53, 98.1%) |  |  |
| ontbijt | √ (47, 87%) |  |  |  |  |
| ontrouw |  |  | √ (52, 96.3%) |  |  |
| ontslag |  |  | √ (47, 85.5%) |  |  |
| onzeker |  |  | √ (47, 85.5%) |  |  |
| oorlog |  |  | √ (50, 92.6%) |  |  |
| ophangen |  |  | √ (25, 46.3%) |  |  |
| oplichten |  |  | √ (45, 84.9%) |  |  |
| opsluiten |  |  | √ (47, 87%) |  |  |
| **opwinden*** | √ (14, 26.9%) |  |  | √ (23, 44.2%) |  |
| orgasme | √ (45, 83.3%) |  |  |  |  |
| orgie |  |  | √ (28, 52.8%) |  |  |
| overleg | √ (36, 65.5%) |  |  |  |  |
| paniek |  |  | √ (50, 94.3%) |  |  |
| papier |  | √ (30, 54.5%) |  |  |  |
| parade | √ (25, 45.5%) |  |  |  |  |
| paragraaf |  | √ (45, 86.5%) |  |  |  |
| paring |  | √ (26, 49.1%) |  |  |  |
| passen |  | √ (22, 40%) |  |  |  |
| pedofiel |  |  | √ (52, 98.1%) |  |  |
| piano | √ (34, 65.4%) |  |  |  |  |
| piekeren |  |  | √ (41, 75.9%) |  |  |
| pijpen | √ (22, 40.7%) |  |  |  |  |
| pilaar |  | √ (42, 79.2%) |  |  |  |
| pinda |  | √ (26, 47.3%) |  |  |  |
| plafond |  | √ (40, 74.1%) |  |  |  |
| plank |  | √ (42, 76.4%) |  |  |  |
| **politie*** | √ (16, 29.1%) |  |  | √ (24, 43.6%) |  |
| portret |  | √ (31, 57.4%) |  |  |  |
| postzegel |  | √ (39, 70.9%) |  |  |  |
| potlood |  | √ (41, 75.9%) |  |  |  |
| priester |  | √ (28, 52.8%) |  |  |  |
| **prijs*** |  | √ (17, 31.5%) |  | √ (19, 35.2%) |  |
| probleem |  |  | √ (29, 54.7%) |  |  |
| profiteur |  |  | √ (46, 85.2%) |  |  |
| programma |  | √ (30, 55.6%) |  |  |  |
| project |  | √ (26, 47.3%) |  |  |  |
| provincie |  | √ (33, 61.1%) |  |  |  |
| pudding | √ (32, 59.3%) |  |  |  |  |
| raadsel |  | √ (24, 44.4%) |  |  |  |
| razernij |  |  | √ (47, 87%) |  |  |
| reden |  | √ (30, 56.6%) |  |  |  |
| regel |  | √ (24, 42.9%) |  |  |  |
| regenton |  | √ (35, 67.3%) |  |  |  |
| register |  | √ (41, 75.9%) |  |  |  |
| reizen | √ (45, 83.3%) |  |  |  |  |
| rendement | √ (25, 47.2%) |  |  |  |  |
| rente | √ (18, 34%) |  |  |  |  |
| reuma |  |  | √ (51, 92.7%) |  |  |
| rijmen | √ (29, 53.7%) |  |  |  |  |
| rillen |  |  | √ (31, 58.5%) |  |  |
| rivier | √ (31, 56.4%) |  |  |  |  |
| roddel |  |  | √ (44, 83%) |  |  |
| roeren |  | √ (39, 72.2%) |  |  |  |
| roofmoord |  |  | √ (54, 98.2%) |  |  |
| rouwen |  |  | √ (34, 61.8%) |  |  |
| ruzie |  |  | √ (49, 90.7%) |  |  |
| sadist |  |  | √ (50, 92.6%) |  |  |
| sandaal |  | √ (22, 41.5%) |  |  |  |
| sarren |  |  | √ (47, 87%) |  |  |
| schaamte |  |  | √ (43, 79.6%) |  |  |
| schaar |  | √ (36, 67.9%) |  |  |  |
| schaden |  |  | √ (49, 92.5%) |  |  |
| schandaal |  |  | √ (48, 87.3%) |  |  |
| schande |  |  | √ (45, 86.5%) |  |  |
| scheiding |  |  | √ (43, 79.6%) |  |  |
| schelden |  |  | √ (48, 85.7%) |  |  |
| schoen |  | √ (31, 57.4%) |  |  |  |
| schoft |  |  | √ (50, 92.6%) |  |  |
| schok |  |  | √ (36, 65.5%) |  |  |
| school | √ (26, 48.1%) |  |  |  |  |
| schreeuw |  |  | √ (32, 59.3%) |  |  |
| schroef |  | √ (43, 78.2%) |  |  |  |
| schuld |  |  | √ (45, 86.5%) |  |  |
| schuren |  | √ (27, 50.9%) |  |  |  |
| schurft |  |  | √ (45, 83.3%) |  |  |
| **seksfilm*** | √ (16, 29.6%) | √ (16, 29.6%) |  |  |  |
| september | √ (24, 43.6%) |  |  |  |  |
| sigaar |  |  | √ (22, 40%) |  |  |
| sigaret |  |  | √ (33, 60%) |  |  |
| slaan |  |  | √ (49, 90.7%) |  |  |
| slaapzaal |  | √ (26, 49.1%) |  |  |  |
| slager |  | √ (29, 53.7%) |  |  |  |
| sleutel |  | √ (31, 58.5%) |  |  |  |
| slijmen |  |  | √ (46, 86.6%) |  |  |
| slipje |  | √ (27, 49.1%) |  |  |  |
| sloot |  | √ (35, 66%) |  |  |  |
| smoking |  | √ (26, 48.1%) |  |  |  |
| snauwen |  |  | √ (53, 98.1%) |  |  |
| sneuvelen |  |  | √ (48, 90.6%) |  |  |
| sperma |  | √ (22, 41.5%) |  |  |  |
| spijten |  |  | √ (25, 46.3%) |  |  |
| spinazie | √ (38, 69.1%) |  |  |  |  |
| spoelen |  | √ (40, 72.7%) |  |  |  |
| sport | √ (39, 73.6%) |  |  |  |  |
| stadhuis |  | √ (28, 50.9%) |  |  |  |
| stank |  |  | √ (50, 90.9%) |  |  |
| steekwond |  |  | √ (50, 94.3%) |  |  |
| steelpan |  | √ (37, 67.3%) |  |  |  |
| steen |  | √ (37, 66.1%) |  |  |  |
| steil |  | √ (34, 63%) |  |  |  |
| stellen |  | √ (37, 67.3%) |  |  |  |
| sterven |  |  | √ (35, 66%) |  |  |
| stier |  | √ (24, 44.4%) |  |  |  |
| stikken |  |  | √ (48, 88.9%) |  |  |
| stoel |  | √ (40, 74.1%) |  |  |  |
| stoelpoot |  | √ (45, 86.5%) |  |  |  |
| stoep |  | √ (45, 81.8%) |  |  |  |
| stomerij |  | √ (40, 74.1%) |  |  |  |
| straat |  | √ (35, 63.6%) |  |  |  |
| straf |  |  | √ (42, 77.8%) |  |  |
| straffen |  |  | √ (31, 57.4%) |  |  |
| straling |  |  | √ (31, 57.4%) |  |  |
| stranden |  |  | √ (18, 34.6%) |  |  |
| stukadoor |  | √ (34, 65.4%) |  |  |  |
| taart | √ (46, 86.8%) |  |  |  |  |
| tafel |  | √ (38, 69.1%) |  |  |  |
| takken |  | √ (38, 69.1%) |  |  |  |
| tapijt |  | √ (34, 63%) |  |  |  |
| tarten |  |  | √ (41, 75.9%) |  |  |
| tarwe |  | √ (26, 48.1%) |  |  |  |
| tegel |  | √ (47, 87%) |  |  |  |
| tegenslag |  |  | √ (45, 84.9%) |  |  |
| tegenzin |  |  | √ (41, 77.4%) |  |  |
| teisteren |  |  | √ (42, 77.8%) |  |  |
| tekenen | √ (31, 57.4%) |  |  |  |  |
| tekort |  |  | √ (46, 88.5%) |  |  |
| telefoon | √ (23, 42.6%) |  |  |  |  |
| telegram |  | √ (36, 65.5%) |  |  |  |
| teller |  | √ (43, 78.2%) |  |  |  |
| termijn |  | √ (30, 56.6%) |  |  |  |
| theedoek |  | √ (37, 67.3%) |  |  |  |
| theelepel |  | √ (47, 85.5%) |  |  |  |
| theorie |  | √ (28, 50.9%) |  |  |  |
| tijdperk |  | √ (39, 72.2%) |  |  |  |
| timmerman | √ (27, 50.9%) |  |  |  |  |
| tiran |  |  | √ (49, 94.2%) |  |  |
| tobben |  |  | √ (44, 80%) |  |  |
| toetje | √ (44, 81.5%) |  |  |  |  |
| toilet |  | √ (24, 43.6%) |  |  |  |
| tongzoen | √ (42, 77.8%) |  |  |  |  |
| traditie | √ (27, 50.9%) |  |  |  |  |
| traject |  | √ (30, 56.6%) |  |  |  |
| trauma |  |  | √ (51, 94.4%) |  |  |
| trede |  | √ (49, 89.1%) |  |  |  |
| treden |  | √ (46, 86.8%) |  |  |  |
| treiteren |  |  | √ (54, 98.2%) |  |  |
| trekken |  | √ (29, 51.8%) |  |  |  |
| treuren |  |  | √ (45, 83.3%) |  |  |
| triest |  |  | √ (50, 90.9%) |  |  |
| trompet |  | √ (24, 45.3%) |  |  |  |
| troosten | √ (44, 81.5%) |  |  |  |  |
| trottoir |  | √ (45, 81.8%) |  |  |  |
| twijfelen |  |  | √ (31, 58.5%) |  |  |
| uitgever |  | √ (40, 74.1%) |  |  |  |
| uitjouwen |  |  | √ (51, 96.2%) |  |  |
| uitlachen |  |  | √ (50, 92.6%) |  |  |
| uitzenden |  | √ (30, 55.6%) |  |  |  |
| urine |  | √ (29, 55.8%) |  |  |  |
| vagina | √ (25, 46.3%) |  |  |  |  |
| vakantie | √ (50, 94.3%) |  |  |  |  |
| varen | √ (40, 71.4%) |  |  |  |  |
| venster |  | √ (32, 58.2%) |  |  |  |
| verband |  | √ (19, 34.5%) |  |  |  |
| verdord |  |  | √ (42, 77.8%) |  |  |
| verdriet |  |  | √ (42, 76.4%) |  |  |
| verdwalen |  |  | √ (44, 83%) |  |  |
| vergelijk |  | √ (31, 58.5%) |  |  |  |
| vergroten |  | √ (30, 56.6%) |  |  |  |
| verlammen |  |  | √ (51, 94.4%) |  |  |
| verlies |  |  | √ (48, 90.6%) |  |  |
| verlinken |  |  | √ (51, 92.7%) |  |  |
| verloren |  |  | √ (49, 89.1%) |  |  |
| vernielen |  |  | √ (49, 92.5%) |  |  |
| verraad |  |  | √ (53, 96.4%) |  |  |
| versie |  | √ (42, 79.2%) |  |  |  |
| vertalen |  | √ (30, 55.6%) |  |  |  |
| verveling |  |  | √ (48, 90.6%) |  |  |
| vervoer | √ (27, 50%) |  |  |  |  |
| verwijten |  |  | √ (48, 88.9%) |  |  |
| verzuipen |  |  | √ (52, 96.3%) |  |  |
| vierkant |  | √ (47, 88.7%) |  |  |  |
| vijand |  |  | √ (48, 90.6%) |  |  |
| vingers |  | √ (26, 48.1%) |  |  |  |
| vlees | √ (24, 45.3%) |  |  |  |  |
| vleugel |  | √ (27, 50%) |  |  |  |
| vliegtuig | √ (30, 54.4%) |  |  |  |  |
| vloer |  | √ (38, 67.9%) |  |  |  |
| vluchten |  |  | √ (35, 63.6%) |  |  |
| vodka |  | √ (28, 51.9%) |  |  |  |
| voertuig | √ (27, 50.9%) |  |  |  |  |
| voetbal | √ (21, 38.2%) |  |  |  |  |
| voetpad |  | √ (34, 64.2%) |  |  |  |
| voorstel | √ (23, 44.2%) |  |  |  |  |
| vrees |  |  | √ (49, 90.7%) |  |  |
| vrijen | √ (48, 87.3%) |  |  |  |  |
| waanzin |  |  | √ (41, 75.9%) |  |  |
| walging |  |  | √ (53, 98.1%) |  |  |
| wandelen | √ (48, 88.9%) |  |  |  |  |
| wanhoop |  |  | √ (52, 98.1%) |  |  |
| wanhopen |  |  | √ (51, 96.2%) |  |  |
| wapen |  |  | √ (41, 73.2%) |  |  |
| water | √ (41, 77.4%) |  |  |  |  |
| weemoed |  |  | √ (29, 53.7%) |  |  |
| weigering |  |  | √ (29, 53.7%) |  |  |
| wereld | √ (26, 48.1%) |  |  |  |  |
| werkeloos |  |  | √ (48, 87.3%) |  |  |
| werken | √ (36, 67.9%) |  |  |  |  |
| winkel | √ (26, 48.1%) |  |  |  |  |
| winkelier |  | √ (33, 60%) |  |  |  |
| winter | √ (21, 38.9%) |  |  |  |  |
| wippen | √ (25, 45.5%) |  |  |  |  |
| woede |  |  | √ (44, 81.5%) |  |  |
| woedend |  |  | √ (45, 83.3%) |  |  |
| wonen | √ (44, 80%) |  |  |  |  |
| woord |  | √ (34, 61.8%) |  |  |  |
| wormen |  | √ (23, 42.6%) |  |  |  |
| wortel |  | √ (28, 51.9%) |  |  |  |
| wraak |  |  | √ (50, 90.9%) |  |  |
| wreedheid |  |  | √ (52, 94.5%) |  |  |
| wurgen |  |  | √ (52, 96.3%) |  |  |
| zakje |  | √ (42, 77.8%) |  |  |  |
| zandloper |  | √ (41, 75.9%) |  |  |  |
| zegel |  | √ (42, 79.2%) |  |  |  |
| zeiken |  |  | √ (48, 88.9%) |  |  |
| zelfmoord |  |  | √ (39, 73.6%) |  |  |
| zetel |  | √ (43, 79.6%) |  |  |  |
| zeuren |  |  | √ (54, 100%) |  |  |
| ziekte |  |  | √ (47, 88.7%) |  |  |
| zielig |  |  | √ (49, 90.7%) |  |  |
| zitplaats | √ (26, 49.1%) |  |  |  |  |
| zoenen | √ (53, 98.1%) |  |  |  |  |
| zondebok |  |  | √ (50, 92.6%) |  |  |
| zonlicht | √ (51, 94.4%) |  |  |  |  |
| zuchten |  |  | √ (25, 47.2%) |  |  |
| zwakheid |  |  | √ (37, 69.8%) |  |  |

*Note*: number of allocations and valid percentage reported; highest percentage reported; in the instance of both positive and negative allocation, the next highest result is reported; *= has variable allocations.

**Appendix E.**

Multi-allocated words with the corresponding highest and second highest valence.

| **Word** | **Allocated valences** |
| --- | --- |
| afwachten | Both positive and negative; negative; neutral |
| alcohol | Both positive and negative; negative |
| geheim | Both positive and negative; negative |
| houding | Both positive and negative; neutral |
| huilen | Both positive and negative; negative |
| kritiek | Both positive and negative; negative |
| lenen | Both positive and negative; negative |
| opwinden | Both positive and negative; positive |
| politie | Both positive and negative; positive |
| prijs | Both positive and negative; neutral |
| seksfilm | Positive; neutral |

*Note*: allocations include the highest percentage first, followed by the next highest result.

**Appendix F.**

Positive valenced words.

| **Positive valence allocations** | | | | | **Removed words*** |
| --- | --- | --- | --- | --- | --- |
| aansporen  aardbei  anker  appel  appelflap  arbeid  architect  badkamer  bakken  banaan  bellen  bespreken  bestaan  borsten  condoom  contact  dagblad  dialect  dineren  diploma  disco  douche  erectie | erotisch  fiets  filmen  flirten  ganzenbord  gebak  gehoor  geweten  gezicht  gitaar  graan  groen  groente  heelal  honing  horloge  kapper  katten  klasse  koffie  korting  kostuum | krant  kruid  kussen  landbouw  leraar  lopen  maken  matras  mening  minnaar  naspel  neuken  minnaar  naspel  neuken  oever  omelet  onderzoek  ontbijt  opwinden  orgasme  overleg | parade  piano  pijpen  politie  pudding  reizen  rendement  rente  rijmen  rivier  school  september  spinazie  sport  taart  tekenen  telefoon  timmerman  toetje  tongzoen  traditie  troosten | vagina  vakantie  varen  vervoer  vlees  vliegtuig  voertuig  voetbal  voorstel  vrijen  wandelen  water  wereld  werken  winkel  winter  wippen  wonen  zitplaats  zonlicht | seksfilm  zoenen |
| *Note*: does not indicate a finalised standardised set of positive words due to no positive words being included at the initial procedure. All positive outcomes are assumed to be false neutral words, due to the lack of a positive word set in the study; *= words removed due to multiple concrete allocations, coefficient of variation fail, and missing values on Cronbach’s alpha analysis. | | | | | |

**Appendix G.**

Neutral valenced words.

| **Neutral valence allocations** | | | | | **Removed words*** |
| --- | --- | --- | --- | --- | --- |
| aandeel  aanmaken  accent  advocaat  afnemen  aftrekken  ambtenaar  antwoord  archief  baard  balkon  balpen  basketbal  bedrijf  beleggen  beleid  bestek  bestuur  biljet  bladzijde  blikje  blinddoek  borduren  boter  broek  brommer  bureau  burger  chauffeur  cirkel  citaat  conclusie  consulaat  dakgoot  deurknop  document  dossier | dubbel  dwerg  eetlepel  eicel  element  elleboog  etiket  faculteit  fietsbel  firma  flacon  fornuis  functie  gebouw  geluid  glaasje  gordijn  grijs  groep  grondstof  haarbos  hagedis  haken  handel  handtas  hebben  heuvel  ijzer  injectie  instituut  ivoor  jarretel  kabinet  kader  kanaal  kantoor  kelder | kenteken  kever  klant  koelkast  komen  kozijn  kraan  laden  laken  legpuzzel  leuning  links  magazijn  meedelen  meester  metaal  meten  methode  metselaar  meubel  middel  naald  octaaf  omroep  papier  paragraaf  paring  passen  pilaar  pinda  plafond  plank  portret  postzegel  potlood  priester  programma | project  provincie  raadsel  reden  regel  regenton  register  roeren  sandaal  schaar  schoen  schroef  schuren  slaapzaal  slager  sleutel  slipje  sloot  smoking  sperma  spoelen  stadhuis  steelpan  steen  steil  stellen  stier  stoel  stoelpoot  stoep  stomerij  straat  stukadoor  tafel  takken  tapijt  tarwe | tegel  teller  termijn  theedoek  theelepel  theorie  tijdperk  toilet  traject  trede  treden  trekken  trompet  trottoir  uitgever  uitzenden  urine  venster  verband  vergelijk  vergroten  versie  vertalen  vierkant  vingers  vleugel  vloer  vodka  voetpad  winkelier  woord  wormen  wortel  zakje  zandloper  zegel  zetel | absorptie  dagblad  dozijn  hertogin  telegram  houding  korting  kostuum  oever  prijs  seksfilm |
| *Note*: *= words removed due to multiple concrete allocations, coefficient of variation fail, and missing values on Cronbach’s alpha analysis. | | | | | |

**Appendix H.**

Negative valenced words.

| **Negative valence allocations** | | | | | **Removed Words*** |
| --- | --- | --- | --- | --- | --- |
| aanklacht  aanslag  aantasten  abortus  afgaan  afgrijzen  afgunst  afhakken  afkeer  afkeuring  afscheid  afschuw  afsnauwen  afval  afwijzing  agressie  angst  argwaan  armoede  asbak  atoombom  bedreigen  beklemmen  belazerd  benauwen  beroerd  beroerte  besmetten  bespotten  betasten  beven  blaam  bloed  bloedbad  bloeden  blunder  boete  bordeel  braaksel  buikpijn  chanteren  conflict  crimineel  crisis  dader  depressie | dodelijk  doden  doodgaan  doodslag  doodsteek  doodstraf  drama  drammen  dreigen  dreun  droefheid  droevig  drugs  duister  dwang  dwingen  ergeren  ergernis  etter  falen  folteren  gebrek  geheim  getreiter  gevaar  gevecht  geweld  gezwel  gifslang  griep  hakmes  haten  hekel  honger  hopeloos  huilen  huiveren  hypocriet  hysterie  inbraak  infectie  instorten  jaloezie  janken  jennen  kadaver | kanker  kerkhof  kermen  klacht  knorrig  kogel  kokhalzen  koorts  kotsen  krenken  krijsen  kritiek  kutje  kwaal  kwellen  lafaard  laster  lastig  lawaai  lenen  lepra  letsel  leugen  levenloos  liegen  lijden  lusteloos  meeloper  messteek  misdaad  misdrijf  miskraam  misleiden  mislukt  mismaakt  misvormen  moeten  monster  moord  nederlaag  noodkreet  noodlot  omkomen  ongeluk  ongeval  onkruid  onlust | ontrouw  ontslag  onzeker  oorlog  ophangen  oplichten  opsluiten  orgie  piekeren  probleem  profiteur  razernij  reuma  rillen  roddel  rouwen  ruzie  sadist  sarren  schaamte  schaden  schandaal  schande  scheiding  schelden  schoft  schok  schreeuw  schuld  schurft  sigaar  sigaret  slaan  slijmen  snauwen  sneuvelen  spijten  stank  steekwond  sterven  stikken  straf  straffen  straling  stranden | tarten tegenslag  tegenzin  teisteren  tekort  tiran  tobben  trauma  treuren  triest  twijfelen  uitjouwen  uitlachen  verdord  verdriet  verdwalen  verlammen  verlies  verlinken  verloren  vernielen  verveling  verwijten  verzuipen  vijand  vluchten  vrees  waanzin  walging  wanhoop  wanhopen  wapen  weemoed  weigering  werkeloos  woede  woedend  wraak  wreedheid  wurgen  zeiken  zelfmoord  ziekte  zielig  zondebok  zuchten  zwakheid | afblaffen  afkraken  afwachten  bedrieger  bedrog  diefstal  ellende  gijzeling  incest  kwetsen  martelen  moedeloos  onmacht  onrecht  paniek  pedofiel  roofmoord  treiteren  verraad  zeuren |
| *Note*: *= words removed due to multiple concrete allocations, coefficient of variation fail, and missing values on Cronbach’s alpha analysis.. | | | | | |

**Appendix I.**

Finalised word sets allocated for a representative valence.

| **Neutral words** | | **Negative words** | | | |
| --- | --- | --- | --- | --- | --- |
| balpen  bestek  bladzijde  blikje  bureau  chauffeur  citaat  consulaat  dakgoot  deurknop  document  eetlepel  elleboog  firma  flacon  gebouw  ijzer  kader  kenteken  kozijn  kraan  leuning  magazijn  middel  paragraaf | pilaar  plank  postzegel  potlood  register  roeren  schroef  spoelen  stoel  stoelpoot  stoep  tegel  teller  theelepel  tijdperk  treden  trottoir  uitgever  versie  vierkant  zakje  zandloper  zegel | aanslag  aantasten  afgaan  afgrijzen  afgunst  afhakken  afkeer  afkeuring  afkraken  afschuw  afsnauwen  afwijzing  agressie  angst  argwaan  armoede  atoombom  bedreigen  beklemmen  belazerd  benauwen  beroerd  beroerte  besmetten  bespotten  beven  bloedbad  bloeden  blunder  boete  braaksel  buikpijn  chanteren  conflict  crimineel  crisis  dader  depressie  diarree  dodelijk  doden  doodgaan  doodslag  doodsteek  doodstraf  drammen  dreigen  dreun  droefheid  droevig | drugs  dwang  dwingen  ergeren  ergernis  etter  falen  folteren  gebrek  getreiter  gevaar  gevecht  geweld  gezwel  gijzeling  griep  haten  hekel  honger  hopeloos  huiveren  hypocriet  hysterie  inbraak  incest  infectie  instorten  jaloezie  jennen  kanker  kermen  klacht  kogel  kokhalzen  koorts  kotsen  krenken  krijsen  kwaal  kwellen  kwetsen  lafaard  laster  lawaai  lepra  letsel  leugen  levenloos  liegen  lijden | lusteloos  martelen  meeloper  messteek  misdaad  misdrijf  miskraam  misleiden  mislukt  mismaakt  misvormen  moedeloos  moord  nederlaag  noodkreet  noodlot  omkomen  ongeluk  ongeval  onlust  onmacht  onrecht  ontrouw  ontslag  onzeker  oorlog  oplichten  opsluiten  paniek  pedofiel  piekeren  profiteur  razernij  reuma  roddel  roofmoord  ruzie  sadist  sarren  schaamte  schaden  schandaal  schande  scheiding  schelden  schoft  schuld  schurft  slaan  slijmen | snauwen  sneuvelen  stank  steekwond  stikken  straf  tarten  tegenslag  tegenzin  teisteren  tekort  tiran  tobben  trauma  treiteren  treuren  triest  uitjouwen  uitlachen  verdord  verdriet  verdwalen  verlammen  verlies  verlinken  verloren  vernielen  verraad  verveling  verwijten  verzuipen  vijand  vrees  waanzin  walging  wanhoop  wanhopen  wapen  werkeloos  woede  woedend  wraak  wreedheid  wurgen  zeiken  zelfmoord  ziekte  zielig  zondebok |

*Note*: Table includes words which have a valid percentage of ≥ 70% in allocation and excludes words which failed the validation checks.
